# RpH-ILV: Probe for lysosomal pH and acute LLOMe-induced membrane permeabilization in cell lines and *Drosophila*

**DOI:** 10.1126/sciadv.adr7325

**Published:** 2025-01-03

**Authors:** Izaak J. Cheetham-Wilkinson, Bhavya Sivalingam, Chloe Flitton, Franziska Flottmann, Luisa Vehling, Maik Drechsler, Marija Stojchevska, Andrea Raimondi, Achim Paululat, Florian Fröhlich, Laura E. Swan, Massimiliano Stagi

**Affiliations:** ^1^ Department of Biochemistry Cell and Systems Biology, Institute of Systems, Molecular and Integrative Biology, University of Liverpool, Liverpool, UK.; ^2^ Division of Molecular Membrane Biology, Department of Biology/Chemistry, Osnabrück University, 49076 Osnabrück, Germany.; ^3^ Department of Zoology & Developmental Biology, Osnabrück University, 49076 Osnabrück, Germany.; ^4^ Experimental Imaging Center, San Raffaele Scientific Institute, Milan, Italy.; ^5^ Università della Svizzera Italiana (USI), Faculty of Biomedical Sciences, Institute for Research in Biomedicine, CH-6500 Bellinzona, Switzerland.; ^6^ Center of Cellular Nanoanalytics Osnabrück – CellNanOs, Osnabrück University, 49076 Osnabrück, Germany.

## Abstract

Lysosomal pH dysregulation is a critical element of the pathophysiology of neurodegenerative diseases, cancers, and lysosomal storage disorders (LSDs). To study the role of lysosomes in pathophysiology, probes to analyze lysosomal size, positioning, and pH are indispensable tools. Here, we developed and characterized a ratiometric genetically encoded lysosomal pH probe, RpH-ILV, targeted to a subpopulation of lysosomal intraluminal vesicles. This subpopulation behaves similarly to the general population of LAMP1-positive vesicles in terms of pH response to pharmacological stresses. In addition, RpH-ILV, which is trafficked to the lysosome via a different cytosolic motif than our previous ratiometric sensor, RpH-LAMP1, is well tolerated by the model organism *Drosophila melanogaster*, exhibits minimal plasma membrane fluorescence, and reveals sensitivity to the lysosomal damaging agent LLOMe, adding a valuable tool to our repertoire of lysosomal pH sensors.

## Teaser

The ratiometric lysosomal probe RpH-ILV enables improved pH tracking and reveals lysosomal dynamics and susceptibility to damage.

## INTRODUCTION

Lysosomal pH is a key metabolic parameter, necessary for mediating ion flux, for tuning the activity of the panoply of lysosomal hydrolases, for regulating the traffic of key transmembrane cargoes to and from the lysosomal membrane, for aspects of lysosomal signaling, and for its fusion to autophagosomal cargoes for digestion and clearance (*1*).

In healthy cells, while lysosomal pH is stable over long periods of time (*2*, *3*), lysosomal pH is maintained by ongoing and dynamic processes (*4*–*9*), and the loss of lysosomal function is communicated rapidly to other organelles (*10*–*13*). In addition, recent studies have shown that lysosomal membrane permeabilization (LMP) is subject to an expanding series of repair mechanisms, which are engaged sequentially as the size and extent of perforations of the lysosomal membrane increase (*14*–*23*). This process of LMP has also been observed in pathological conditions such as cancers, where lysosome-dependent cell death depends on release of lysosomal hydrolases from perforated lysosomes (*24*, *25*) and pathological conditions such as alpha-synuclein aggregation (*26*) and 1-methyl-4-phenyl-1,2,3,6-tetrahydropyridine administration (*27*), suggesting its relevance to neurodegenerative disorders such as Parkinson’s disease.

Therefore, the ability to accurately measure lysosomal pH in response to stimuli and to be able to determine when and how lysosomal pH begins to collapse in response to genetic or chemical stress is of great value to cell biologists and those interested in disease physiology.

We previously built a ratiometric sensor, RpH-3xFLAG (*2*), which is based on a luminal fusion of a mCherry-pHluorin tandem fluorophore to the lysosomal marker protein, lysosomal-associated membrane protein 1 (LAMP1), and a later version, R2pH-3xFLAG, with a 2xmCherry-pHluorin luminal tag (*3*). For clarity, these will be referred to as RpH-LAMP1 and R2pH-LAMP1, respectively. These fusions recapitulate the traffic of endogenous LAMP1, being trafficked to lysosomes from both the transgolgi-late endosome, and the plasma membrane, which leaves a small proportion of unquenched pHluorin fluorescence visible on the plasma membrane, rendering cell sorting via fluorescence-activated cell sorting more technically challenging.

Therefore, we sought to improve several aspects of our probe to provide a complementary mode of lysosomal pH screening with the aim of generating a second lysosomal pH probe that (i) was presented minimally on the plasma membrane, (ii) was trafficked to lysosomes using a different trafficking motif to the LAMP C-terminal YXXØ-based motif, and was (iii) minimally affected lysosomal physiology.

We generated a minimally interactive, genetically encoded lysosome-targeted ratiometric probe based on the transmembrane and cytosolic domains of transmembrane protein 192 (TMEM192), a protein often used for lysosomal tagging (*28*), while replacing the two lysosome-luminal domains of the protein with either pHluorin or mCherry. This probe is found to be enriched in intraluminal vesicles (ILVs) of a large subset of lysosomes, where it strongly colocalizes with the ILV marker protein cluster of differentiation 63 (CD63). These lysosomes behaved indistinguishably from the wider population of LAMP1-positive lysosomes, responding similarly to LAMP1-labeled lysosomes to exogenous dyes and pharmacological treatments known to cause lysosomal stress.

We found that this new probe also provided two unexpected advantages over our LAMP1-based system. RpH-ILV is robustly expressed by *Drosophila* Schneider 2 (S2) cells and can be expressed in transgenic larvae, opening the way to systematic genetic studies of pH in this model. In addition, we found that unlike R2pH-LAMP1, which provides resistance to both l-leucyl-l-leucine methyl ester (LLOMe)–mediated and glycyl-l-phenylalanine 2-naphthylamide (GPN)–mediated membrane permeabilization, lysosomes expressing RpH-ILV remain sensitive to both lysosomal permeabilizing agents, which allows further use of RpH-ILV to investigate the process of transient lysosomal damage and repair.

## RESULTS

### The design of a minimally interactive ratiometric lysosomal probe

**Fig. 1.**
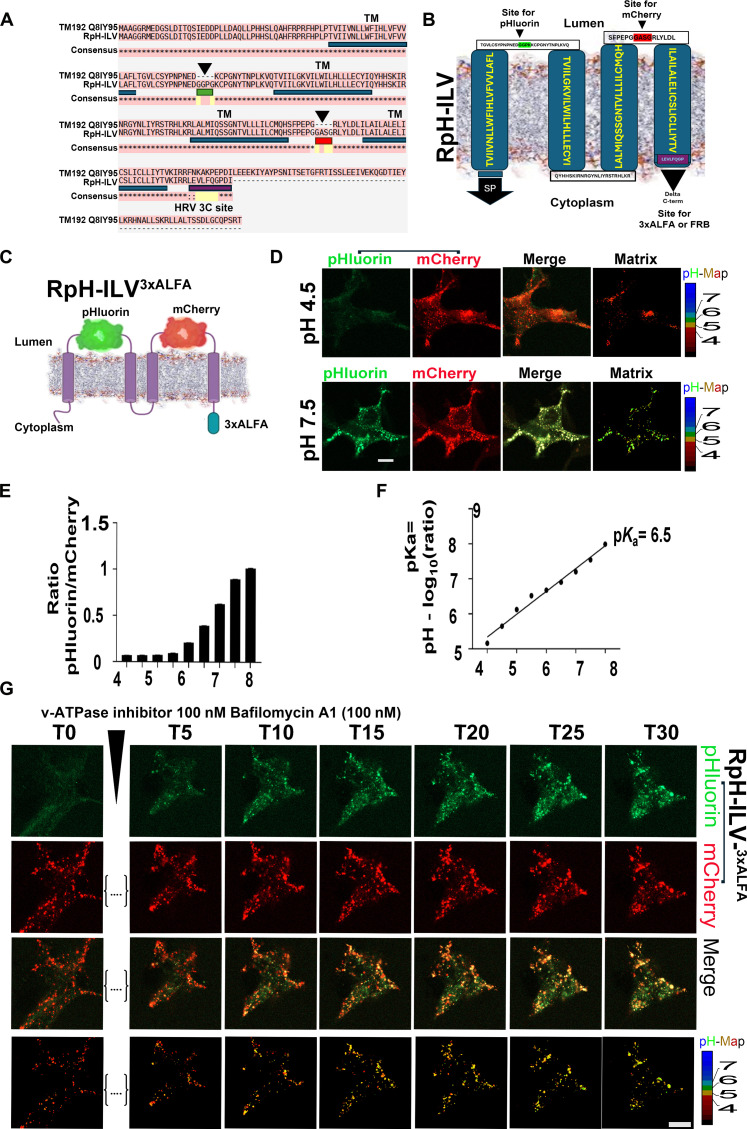
Design of RpH-ILV^3xALFA^ probe. (**A**) Comparison of RpH-ILV sequence against human TMEM192 (UniProt: Q8IY95), showing transmembrane regions (TMs; blue bar), insertion point of pHluorin (arrowhead, green bar), mCherry (arrowhead, red bar), and HRV3C cleavage site (purple bar). N-terminal dileucine motifs are indicated by brackets. (**B** and **C**) Topological representation of RpH-ILV^3xALFA^, including amino acid sequence and cartoon of RpH-ILV^3xALFA^. (**D**) Expression of RpH-ILV^3xALFA^ in HEK293T cells, fixed, permeabilized, and exposed to buffers of defined pH. At pH 4.5, pHluorin is quenched, and at pH 7.5, the pHluorin fluorophore is revealed to colocalize with mCherry fluorescence*.* Scale bar, 10 μm. (**E**) Calibration of ratiometric fluorescence from RpH-ILV^3xALFA^–expressing cells. (**F**) Calculation of p*K*_a_ for RpH-ILV^3xALFA^ probe. (**G**) Use of RpH-ILV^3xALFA^ to measure pH in living cells. Inhibition of lysosomal v-ATPase by 100 nM BafA1 shows rapid unquenching of lysosomal pHluorin fluorescence. T0 (before addition of BafA1) and T5 (5 min after addition of BafA1). Scale bar, 10 μm.

To design a complementary probe to our existing series of LAMP1-based ratiometric probes, we turned to another well-tolerated lysosomal protein, TMEM192, which requires either of two N-terminal dileucine (DxxLL) motifs (LL23/24 and LL28/29; Fig. 1, A to C) for trafficking to the late endosomal/lysosomal compartment (*29*). We aimed to build a construct that trafficked to lysosomes but interacted minimally with other lysosomal resident proteins. We inserted a pHluorin and an mCherry fluorophore in the first and second luminal domains of this protein, respectively. We also truncated the cytosolic C terminus of the protein and replaced it with a recombinant type 14 3C protease from human Rhinovirus (HRV3C) cleavage site and 3xALFA immunoaffinity tag (*30*) for immunolocalization and immunoprecipitation. We named this construct RpH-ILV.

We transfected this construct in human embryonic kidney (HEK) 293T cells (Fig. 1D), where we observed a strong punctate mCherry signal, and minimal pHluorin fluorescence in any compartment, consistent with localization to an acid organelle such as the lysosome. When cells were fixed, permeabilized, and exposed to a buffer pH of 4.5, there was minimal fluorescence from the pHluorin channel, as expected (Fig. 1D). When buffer pH was elevated to pH 7.5, the lysosomal pool of fluorophore was unquenched, revealing pHluorin fluorescence in these punctae. Calibration experiments (*2*) performed in fixed HEK293T cells (Fig. 1, D and E) showed that this probe responded to pH with a p*K*_a_ (where *K*_a_ is the acid dissociation constant) of 6.5. In live HEK293T cells, stably transduced with an inducible RpH-ILV construct, untreated cells showed punctate mCherry fluorescence with minimal pHluorin fluorescence, indicative that our construct traffics to an acidic compartment, as would be expected of a lysosomal probe (Fig. 1G). We then tested a variety of other cell types, which also showed a punctate distribution in the mCherry channel where the pHluorin was quenched, indicative of acid lysosomes (fig. S1). Treatment of live HEK293T cells with the vesicular adenosine triphosphatase (v-ATPase) inhibitor bafilomycin A1 (BafA1) (100 nM) showed a rapid unquenching of the pHluorin fluorophore (Fig. 1G). Treatment of other cell types in live imaging with a combination of BafA1 (100 nM) and the pore forming toxin nigericin (10 μM) (*2*, *31*) causes rapid unquenching of lysosomal pHluorin, confirming the functionality of RpH-ILV in a diverse range of cell types (fig. S2 and movie S1). We also tested RpH-ILV in primary neuronal cultures, where cells were transfected with RpH-ILV on 7 days in vitro (DIV) and imaged at DIV 15. Here, we found strong labeling of acid compartments (fig. S3), which was unquenched by 25-min BafA1/nigericin treatment (fig. S3, B and F, and movie S2). We saw typical behavior of neuronal lysosomes using this construct, where a substantial proportion of lysosomes were stationary in neuronal processes, and a smaller fraction of mobile, acid, vesicles moved both anterogradely and retrogradely in neuronal processes (fig. S3, C and D, and movie S3) (*32*).

### The RpH-ILV probe strongly colocalizes with lysosomal markers

**Fig. 2.**
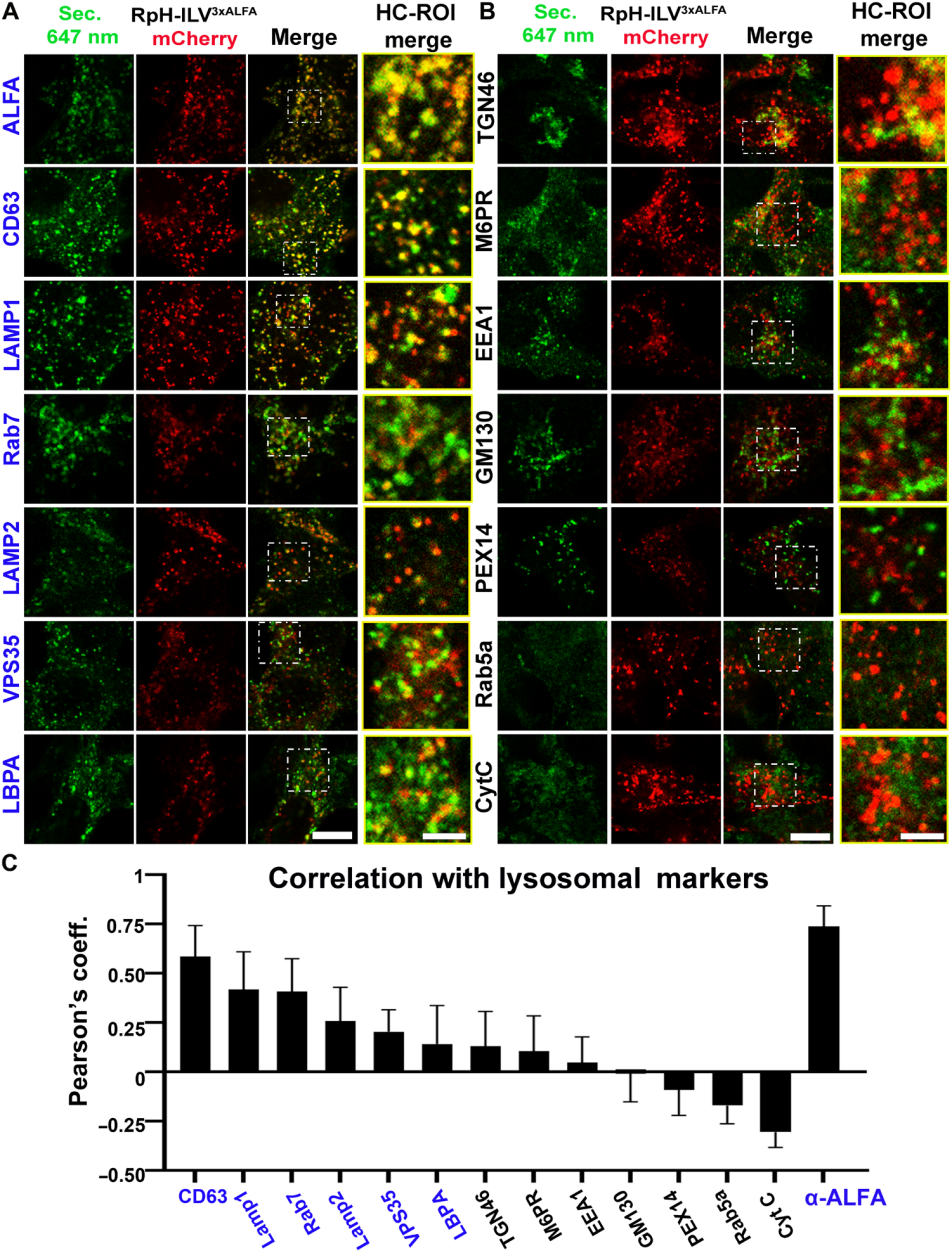
RpH-ILV^3xALFA^ probe colocalises with lysosomal markers. (**A**) Immunostains of a panel of endolysosomal markers (secondary antibody, Alexa 647, green) against mCherry fluorescence from the RpH-ILV^3xALFA^ probe (red). HC-ROI, region of interest with exposure adjusted for contrast. (**B**) Immunostains of a panel of nonlysosomal markers including early endosomal markers such as rab5a and EEA1. Scale bars, (A and B) 10 μm. Scale bar, 5 μm (in HC-ROI). (**C**) Pearson’s correlation coefficient calculated for mCherry versus immunostain RpH-ILV^3xALFA^ is most correlated with itself (ALFA antibody stain) and CD63. Quantification of Pearson’s coefficient in three to four biological replicates per sample, 10 to 30 fields of view per biological replicate (total of 32 to 92 replicates per sample), Welch’s *t* test.

To determine whether our probe had indeed trafficked correctly to lysosomes, we performed a panel of immunostains on a stable HEK293T cell line, inducibly expressing RpH-ILV and fixed with 4% paraformaldehyde (PFA). We incorporated lysosomal makers (Fig. 2A) and non-lysosomal markers (Fig. 2B) and calculated their colocalization via Pearson’s correlation coefficient in four biological replicates per immunostain (Fig. 2C). As expected, the probe correlated most strongly with itself (anti-ALFA tag immunostain; Fig. 2A), followed by the lysosomal marker proteins CD63 and LAMP1. While the overlap with CD63 was nearly complete, a number of LAMP1^+^ punctae were not labeled with the RpH-ILV probe. RpH-ILV also colocalized with Rab7. Given the strong colocalization of RpH-ILV with CD63 in HEK293T cells, we tested whether this colocalization persists in other cell types. We immunostained transiently transfected non-neuronal (fig. S4A) and neuronal (fig. S4B) cells and found that the colocalization of RpH-ILV and CD63 persisted in these cell types. Intriguingly, in some cell types, such as Cos7, which expressed very high levels of CD63 immunoreactivity, RpH-ILV fluorescence was restricted to a subset of CD63-positive puncta but retained its colocalization.

### The RpH-ILV probe colocalizes with markers of functional lysosomes

**Fig. 3.**
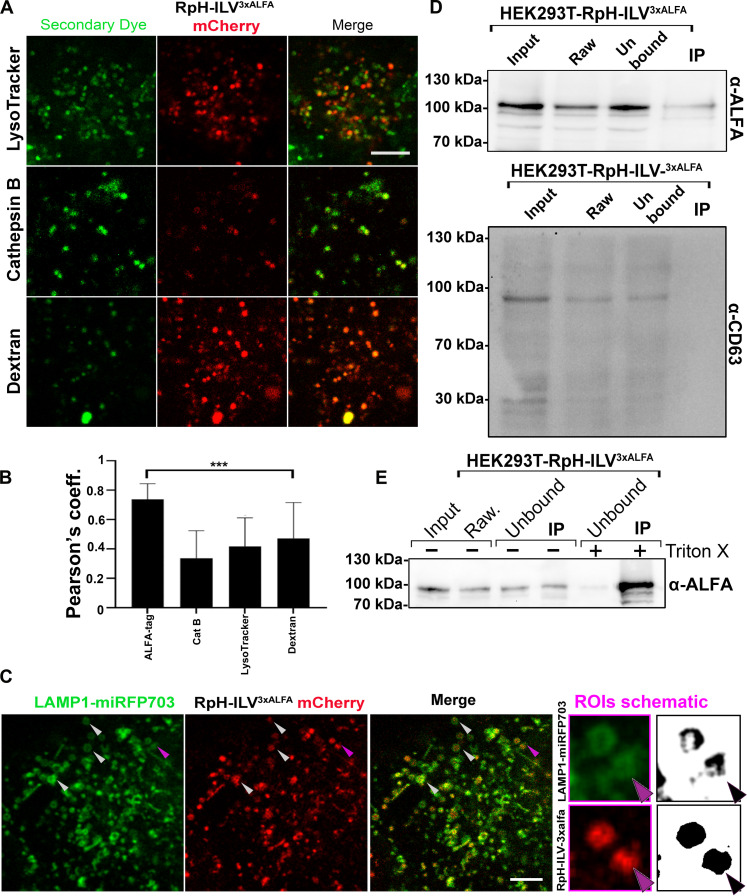
RpH-ILV^3xALFA^ probe colocalises with a subset of active lysosomes. (**A**) Live-cell imaging of HEK293T expressing RpH-ILV^3xALFA^ and labeled with LysoTracker DND-22 Blue (50 nM, 1 hour), the fluorogenic cathepsin B substrate CATB FAST 680 (2 μM, 16 hours), and the fluid-phase dye Alexa647-dextran (20 μg/ml, 16 hours). Dye staining from these probes is represented in green. Scale bar, 5 μm. (**B**) Pearson’s correlation coefficient calculated for mCherry versus probe in four biological replicates of the live dye experiments shown in (A) (6 to 16 fields of view per biological replicate; total of 26 to 92 images per sample, Welch’s *t* test). (**C**) Coexpression of RpH-ILV^3xALFA^ (red, image from mCherry fluorescence) with the broader lysosomal marker LAMP1-mIRFP703 (infrared, represented in green). Arrowheads indicate large LAMP1 positive lysosomes where RpH-ILV^3xALFA^ appears inside a ring of LAMP1 fluorescence. Purple arrowhead: Region indicated in ROI. ROI: Two lysosomes where LAMP1 fluorescence appears as a ring around RpH-ILV^3xALFA^ fluorescence. Scale bar, 5 μm. (**D**) Purifies poorly from whole lysosomal fraction in a stable inducible HEK293T cell line. Input, total cellular fraction; raw, total organelle fraction recovered by freeze lysis; unbound, supernatant after immunoprecipitation (IP). Fractions were also blotted for the strongly colocalizing protein, CD63. The immunoprecipitation fraction did not contain any detectable CD63. (**E**) RpH-ILV^3xALFA^ is readily immunoprecipitated from organelles treated with detergent. Total lysates and organelle fraction were generated as in (D) and then split in two. One fraction was treated with 0.1% (v/v) Triton X-100, and both were immunoprecipitated with ALFA nanobody beads. A substantial fraction of RpH-ILV^3xALFA^ was recovered from detergent-treated organelles.

To confirm that the lysosomes labeled by RpH-ILV were functional, we applied three live-cell markers of lysosomal function, LysoTracker DND-22 Blue, the fluorogenic cathepsin B substrate CATB FAST 680 and the fluid-phase dye Alexa Fluor 647–dextran (Fig. 3A) to cells stably expressing an inducible RpH-ILV construct. All three markers were found enriched in the probe-marked compartment (Fig. 3B), indicating that these lysosomes were functional. We noted, however, that there were several punctae for each of these dyes (LysoTracker, cathepsin B, and dextran), which were not positive for our probe. This suggests that there are functional lysosomes that are not labeled using our RpH-ILV construct, consistent with the immunofluorescence results in Fig. 2.

Next, we compared the distribution of RpH-ILV in live cells with overexpressed far-red fluorescent LAMP1^miRFP703^ (Fig. 3C), where we noticed two interesting features: RpH-ILV colocalized with LAMP1^miRFP703^, but there were more LAMP1 puncta than mCherry-positive probe puncta. In addition, in larger lysosomes (Fig. 3C, arrowheads), it was evident that while LAMP1 formed a ring around the edge of the lysosome, our probe formed smaller punctae inside the LAMP1-positive ring. We suspected that, consistent with its strong colocalization with CD63, our probe may be trafficked to ILVs inside lysosomes. If that were the case, then we would expect that the topologically cytosolic 3xALFA immuno-tag would not be accessible to cytosol. To test this, we performed immunopurifications of organelles (*2*) harvested from stable HEK293T inducibly expressing RpH-ILV (Fig. 3D). While RpH-ILV is readily detectible in the total cell pellet (input) and the raw lysosomal fraction (raw), a very small proportion of the RpH-ILV can be immunoprecipitated from the whole organelle fraction and that small fraction does not appear to enrich its most colocalized lysosomal marker, CD63. However, if we expose our fraction of extracted organelles to the detergent Triton X-100 (0.1%) (Fig. 3E), then the ALFA tag becomes available for immunoprecipitation, and our probe is strongly depleted from the organelle fraction lysates. This led us to believe that most of the RpH-ILV construct is enriched in lysosomal ILVs.

**Fig. 4.**
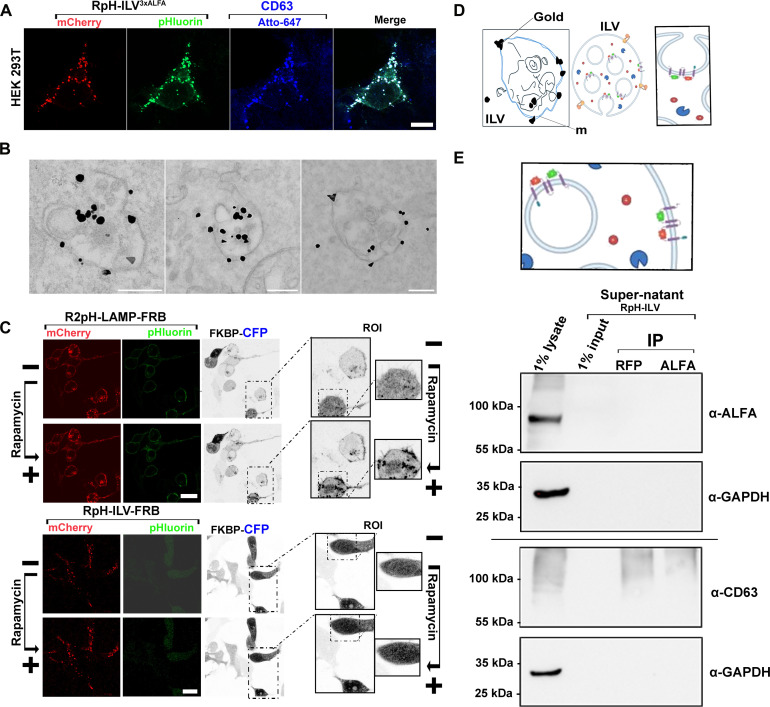
RpH-ILV^3xALFA^ appears to be found on ILVs. (**A**) Colocalization of RpH-ILV^3xALFA^ with CD63 over a whole cell. Scale bar, 5 μm. (**B**) Immunogold labeling of RpH-ILV^3xALFA^ shows that immunolabel is associated with MVB-containing organelles. Scale bar, 200 nm. (**C**) Recruitment of cytosolic CFP using rapamycin inducible dimerization of FRB (on C terminus of ratiometric probe) and FKBT on cytosolic CFP shows that the C terminus of RpH-ILV^3xALFA^ is not exposed to the cytosol, whereas the C-terminal FRB on a LAMP1 ratiometric probe is able to recruit cytosolic proteins. Scale bars, 15 μm. (**D**) Cartoon showing the likely the topology of the RpH-ILV probe. (**E**) Cartoon showing likely the topology of the RpH-ILV^3xALFA^ probe in ILVs and precipitation from conditioned media from 5-hour 200 nM BafA-treated RpH-ILV–inducible HEK293T using the topologically “outside” mCherry on RFP-trap beads and the topologically “inside” ALFA tag on ALFA-PE nanobody beads. Neither antibody was able to enrich RpH-ILV from the media, suggesting that the probe is not appreciably secreted in these conditions. However, CD63 was unspecifically enriched from conditioned media. GAPDH, glyceraldehyde-3-phosphate dehydrogenase.

In HEK293T, RpH-ILV almost totally overlaps with endogenous CD63 (Fig. 4A). To confirm this both on an ultrastructural and topological level, we performed immunogold labeling against the ALFA tag on saponin-permeabilized HEK293T inducibly expressing our probe (Fig. 4B) This revealed that RpH-ILV was found in lysosomes enriched in ILVs. Gold particles were localized both on the cytosolic side of lysosomal limiting membrane (arrows) or were associated with internal ILVs (arrowheads). Despite treatment with saponin, which partially permeabilizes the lysosomal membrane, the immunogold reagents are more accessible for the small fraction of our sensor, which is on the lysosomal limiting membrane compared to the larger proportion that is found on ILVs, but topologically inside the ILV membrane, it is likely that immunogold labeling underestimates the relative proportion of RpH-ILV that is found on ILVs.

To confirm that most of the RpH-ILV is indeed topologically inaccessible to the cytosol, we compared the topology of our LAMP1-based R2pH probe (*2*, *3*) with RpH-ILV. In each case, we replaced the “cytosolic” 3xaffinity tag with the FKBP12-rapamycin-binding (FRB) dimerization motif (Fig. 4C). We then expressed these probes alongside a rapamycin-recruitable cytosolic FK506-binding protein–cyan fluorescent protein (FKBP-CFP) construct. The LAMP1-based R2pH-FRB construct showed its usual distribution [lysosomes (pHluorin quenched) and plasma membrane (pHluorin unquenched)], and the addition of rapamycin rapidly caused recruitment of the FKBP-CFP fluorophore to both compartments. However, we found that RpH-ILV-FRB trafficked to lysosomes only (pHlourin fluorescence quenched) but could not recruit cytosolic FKBP-CFP to this compartment in the presence of the rapamycin dimerising agent, which is consistent with the model that this probe is trafficked to a subset of lysosomes containing ILVs with only a minor fraction present on the lysosomal limiting membrane and thus accessible to cytosolic proteins (modeled in Fig. 4D). To explore whether RpH-ILV is present not only on the same lysosomes as CD63 but on the same ILVs as CD63, we attempted to purify RpH-ILV from cellular supernatants under conditions known to favor secretion of CD63 (*33*). We took conditioned media from cells exposed to 5-hour 200 nM BafA1, which potentiates the secretion of CD63 in HEK293T cells, and attempted to immuno-enrich ILVs marked with RpH-ILV. If secreted, then the luminal mCherry fluorophore will be exposed topologically, while the C-terminal ALFA tag will remain protected in the lumen of the ILV (Fig. 4E). We used red fluorescent protein (RFP) trap and ALFA nanobody beads to probe conditioned media, where we were unable to detect any RpH-ILV secretion, but we were able to unspecifically enrich CD63, suggesting that despite similar location, RpH-ILV does not decorate CD63-positive vesicles that are secreted.

### RpH-ILV interacts minimally with other lysosomal proteins

**Fig. 5.**
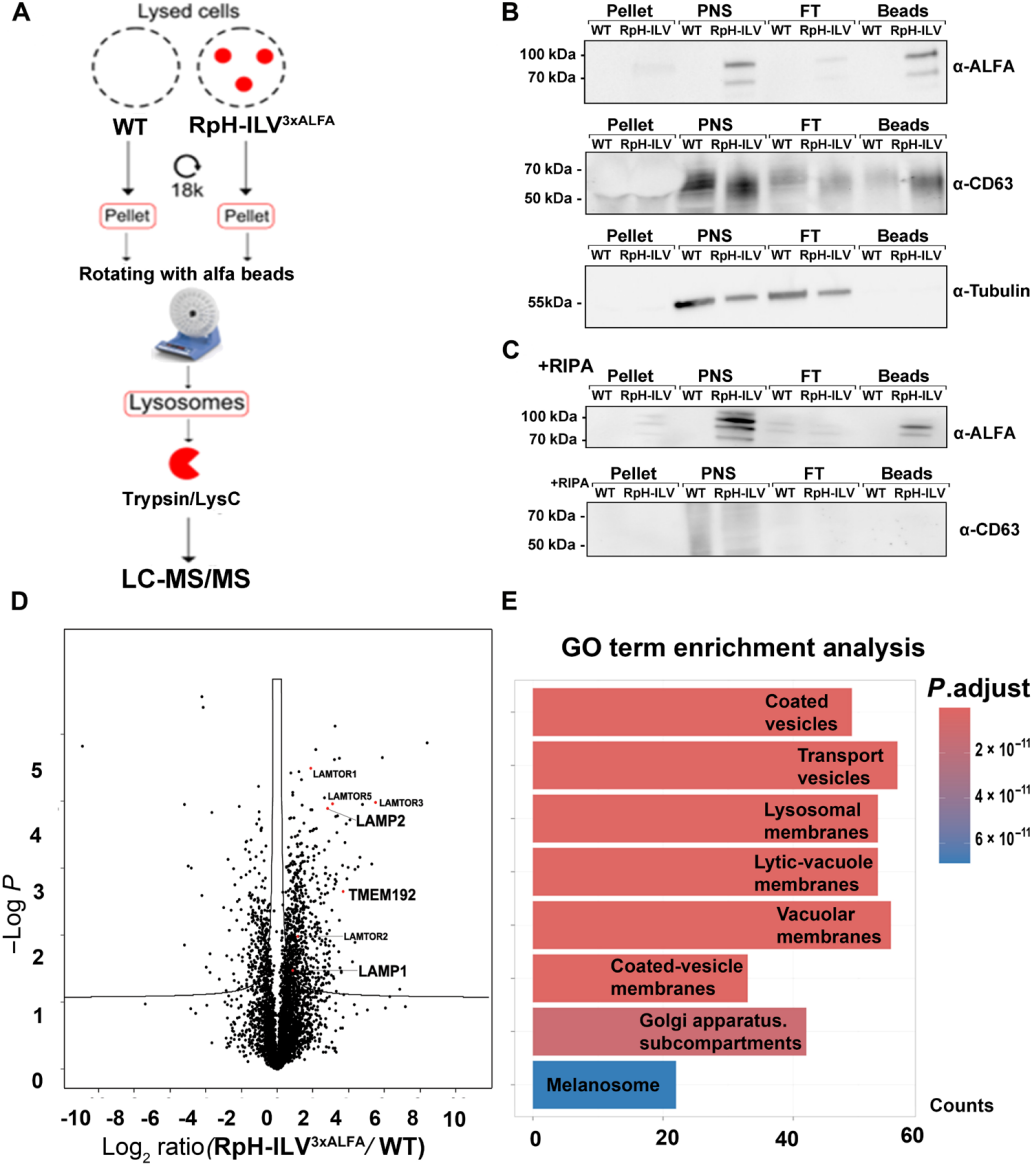
RpH-ILV^3xALFA^ immunoprecipitation enriches lysosomal organelle markers, but not CD63. (**A**) Schematic of immunoprecipitation. HEK293T cells were lysed by dounce homogenization. (**B**) Total cell pellet (pellet), post-nuclear supernatant (PNS), PNS after immunodepletion by anti-ALFA resin [flow through (FT)] (all 1.5% of total), and immune-enriched fraction (25% of total) in detergent-free conditions. (**C**) Immunopurification of RpH-ILV^3xALFA^ in RIPA buffer. Pellet, PNS, and FT all 1.5% of total, and beads 25% of total. (**D**) Volcano plot of proteins identified in anti-ALFA organelle (detergent-free) pulldowns of RpH-ILV^3xALFA^-expressing cells and control (see also Table 1). Key lysosomal markers (LAMTOR1/5/3 LAMP2, TMEM192/RpH-ILV^3xALFA^, LAMTOR2, and LAMP1) are indicated in red. (**E**) GO term analysis of immunopreciptates indicating the enrichment of proteins associated with lysosomes and membrane trafficking. WT, wild type; LC-MS/MS, liquid chromatography tandem mass spectrometry.

Lysosomes from transfected and untransfected cells were isolated via ALFA immunoprecipitation from dounce homogenized cells (Fig. 5A) and analyzed by Western blot or by mass spectrometry–based proteomics. Western blot analysis revealed that RpH-ILV could be enriched, although, as previously (Fig. 3D), a large amount is lost during the precipitation (Fig. 5B). However, the amount of purified lysosomes was sufficient for proteomic analysis. To determine whether RpH-ILV still interacted with proteins known to interact with TMEM192, we first determined whether potential TMEM192 interactors for which peptides were detected were enriched in this pulldown. A total of 4633 proteins were detected in this analysis, and 185 human proteins are labeled as potential TMEM192 interactors in BioGRID (*34*) using the broadest description, which includes whole organelle pulldowns and proximity biotinylation experiments. A total of 114 of these potential interactors were measured in our analysis. Of the 635 proteins enriched, 39 potential interactors of TMEM192 were detected. The majority (75 proteins) were detected but not enriched (Table 1), suggesting that RpH-ILV is, as designed, not enriching proteins that bind to the full-length TMEM192 protein. We did not find that RpH-ILV associated with CD63 in detergent-containing buffers [radioimmunoprecipitation assay (RIPA) buffer; Fig. 5C]. We then analyzed what proteins were enriched in RpH-ILV pulldowns in intact organelles. This analysis revealed that several lysosomal proteins were coenriched with RpH-ILV (Fig. 5D). In addition, Gene Ontology (GO) enrichment analysis was performed to categorize the enriched proteins in the lysosomes of RpH-ILV cells (Fig. 5E). Despite strong colocalization (Figs. 2 and 4), CD63 was not coenriched in the fraction of organelles with surface-presented RpH-ILV probe (Fig. 5, B and D, and Table 1), suggesting that the portion of immune-precipitated structures containing surface RpH-ILV are likely to be earlier endolysosomal structures where CD63 has not been internalized to ILVs via endosomal sorting complex required for transport (ESCRT).

**Table 1.** Putative interactors of full-length TMEM192 detected in RpH-ILV organelle pulldowns by mass spectroscopy. Full list of proteins (retrieved from BioGRID) that directly or indirectly associate with TMEM192. A number of lysosomal proteins were detected and enriched, but CD63 (bold) was not detected in detergent-free RpH-ILV^3xALFA^ immunoprecipitations.

TMEM192 interactors enriched	TMEM192 interactors detected but not enriched	TMEM192 interactors not detected
acp2 app arl8b asph atp6v0d1 atp6v1b2 bms1clta ctsd erlin1 gaa gba ggh glb1 gns golm1gys1 hexb jak1 lamp1 lamp2 lamtor1 lamtor2 lamtor3 mblac2 npc1 pi4k2a pld3 ppt1 rab14 rab5b rab9a scarb2 sdcbp spns1 stom tmem106b tpp1 yars	abhd17b apob arl8a atp6ap1 atp6v1e1 atpaf1 b4galt7 b4gat1 cnp cox6b1 ctsa ctsz cyb5b dnase2 eif2b5 ergic3 ero1l extl3 flot1 flot2 fth1 fyn galns glg1 gnl3 gtpbp4 gusb heatr3 ifi30 ldlr lemd3 man2b1 mmab mrpl15 mrpl30 mrpl34 myo1b napa ndc1 ndufa6 ndufb6 ndufs7 ngdn nipsnap3a npc2 pdcd6 plbd2 ppap2c prcp rab21 rab5c rab7a rhob rpl14 rpl7a saal1 sdc2 sec61a1 slc12a2 slc2a1 slc38a2 slc38a7 sqstm1 stk11ip stx4 stx8 stxbp3 tab1 tfrc tmem59 tomm20 tspan6 usp1 usp9x vrk2 xab2	actr6 atp6v1b1 bola3 bre ccdc114 cd226 cd28 **cd63** cd83 clec12b coa6 ctsc cyr61 efna5 eva1c foxk1 gcsh gprc5a gypa hagh hist1h2aj hist2h2ab hist2h2be icam3 il2ra il7r klrg2 lamp3 lgals9 lilrb3 map1lc3b map3k7 mapk8ip3 mppe1 ndfip1 nsun4 nt5e ntrk3 paep pcdha12 ppt2 ptprh pwwp2a ramp3 rnf149 rnf166 rtp2 sdc4 sec61a2 sema4a slc25a10 slc2a3 slc39a4 smad1 smad3 smad5 syf2 syne4 sypl2 tab2 tmem154 tnfrsf10c tnfrsf9 trdn tspan15 tspan5 ttyh1 ube2d2 vamp5 vkorc1 zdhhc12

### RpH-ILV marked lysosomes respond to lysosomal stressors in the same manner as R2pH-LAMP1–positive lysosomes

**Fig. 6.**
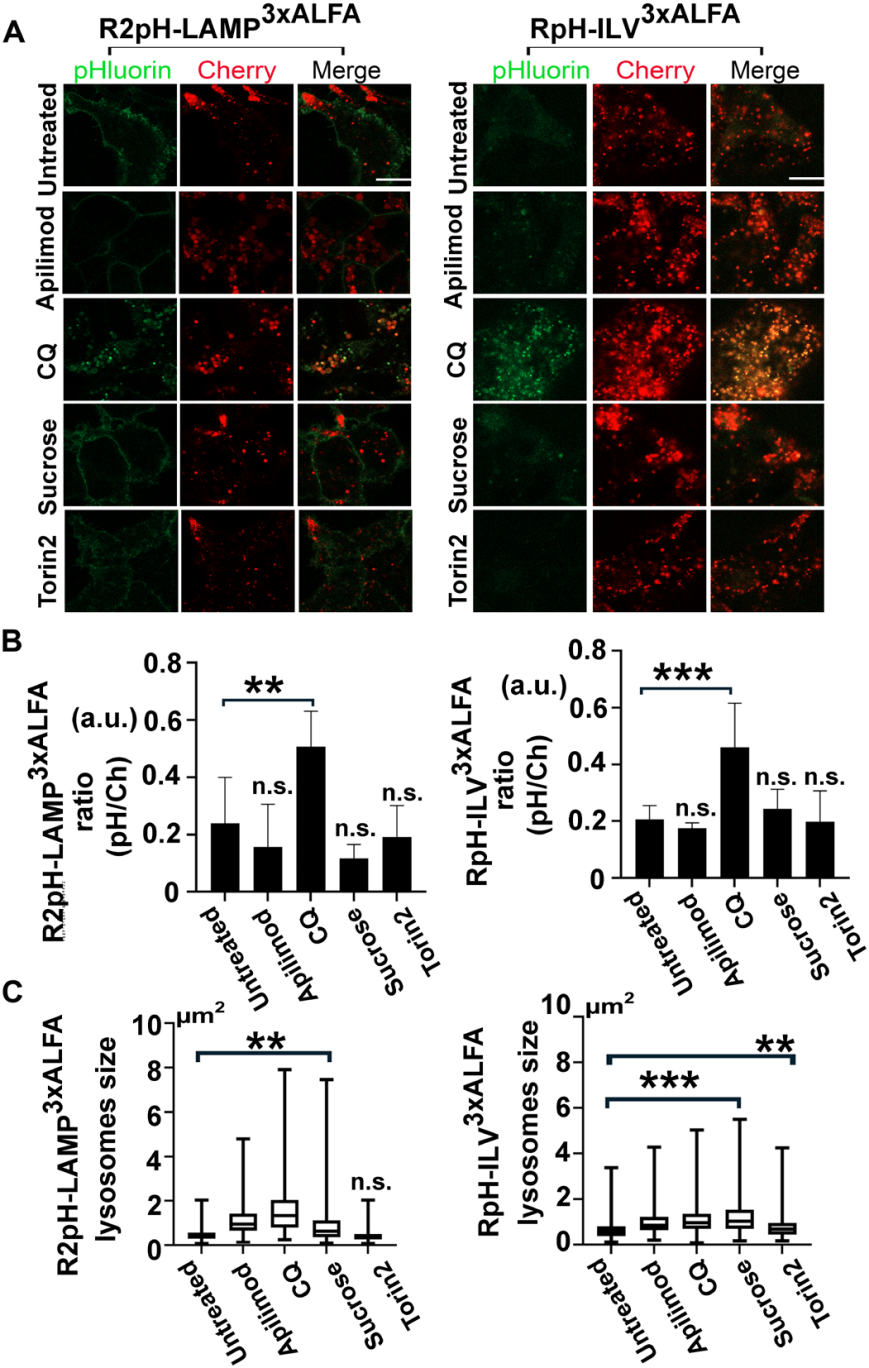
RpH-ILV^3xALFA^ and R2pH-LAMP^3xALFA^ probes respond similarly to pharmacological stressors. (**A**) Stable inducible RpH-ILV^3xALFA^ and stable R2pH-LAMP^3xALFA^ cell lines were exposed to compounds with known effects on lysosomes: apilimod: 3 hours, 20 nM; chloroquine (CQ): 5 hours, 100 μM; sucrose: 4 hours, 90 mM; torin2: 2 hours, 250 nM; all 37°C and 5% CO_2_. Scale bar, 10 μm. (**B**) Quantification of change in ratiometric fluorescence in triplicate samples treated with the compounds listed in (A). (**C**) Measurement of average lysosomal size under each treatment, three to four biological replicates per condition, minimum of 12 fields of view per replicate and 20 to 50 puncta per field of view. Quantification was performed blinded to treatment and probe. a.u., arbitrary unit.

As we had now established that our RpH-ILV probe was found in a subset of LAMP1-positive lysosomes, most likely within ILVs, we tested to see if this subpopulation of lysosomes behaved any differently from the overall population in response to lysosomal stressors. Using a panel of drug treatments (torin2: 2 hours, 250 nM; apilimod: 3 hours, 20 nM, sucrose: 4 hours, 90 mM, chloroquine: 5 hours, 100 μM; all at 37°C with 5% CO_2_) that affect lysosomal function (*2*), we measured the lysosomal pH and lysosomal size after treatment in biological triplicates stably expressing either R2pH-LAMP1^3xALFA^ or inducibly expressing RpH-ILV^3xALFA^ (Fig. 6A). Despite RpH-ILV being present in a smaller subset of lysosomes than R2pH-LAMP, this population exhibited the same changes in pH as the wider, LAMP1-labeled population (Fig. 6B). Measuring the area of sensor-labeled lysosomes after drug treatment, size changes were broadly similar, but the apparent size of R2pH-LAMP objects tended to extend in a greater range, potentially because the R2pH-LAMP probe is found on the lysosomal limiting membrane, whereas RpH-ILV is limited to the lumen of lysosomes.

### RpH-ILV^3xALFA^ is expressed in lysosomes in *Drosophila* S2 cells

**Fig. 7.**
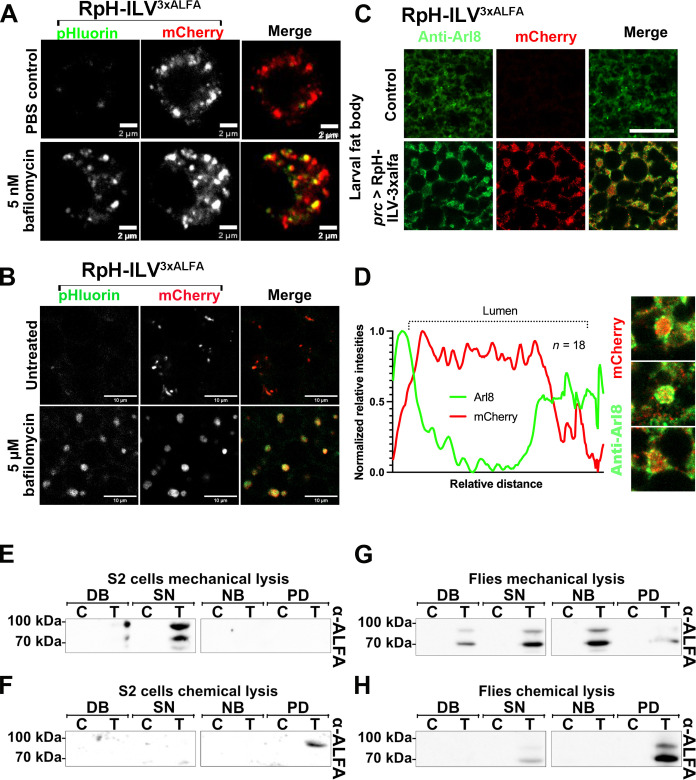
RpH-ILV^3xALFA^ expression in *Drosophila melanogaster*. In transfected *Drosophila* S2 cells (**A**) and transgenic flies (**B**), RpH-ILV^3xALFA^ localizes in acidic organelles where pHluorin fluorescence was quenched (top). The pHluorin signal was only visible after treating the cells with 5 nM proton pump inhibitor BafA1 for 45 min (bottom). The pHluorin channel (green), the mCherry channel (red), and merged images are shown. Dissected third instar larvae of control flies (*white*^1118^, top) and transgenic flies (bottom) were stained with anti-Arl8 antibody (green) as a marker for lysosomes, visualizing the RpH-ILV^3xALFA^ sensor (red) (**C**). Scale bars, (A) 2 μm and (B and C) 0 μm. (**D**) Line intensity plot of the intensity of the Arl8 signal (green) and RpH-ILV (red), indicating a luminal localization of the RpH-ILV^3xALFA^ sensors. Representative Western blots show immunopurifications of RpH-ILV^3xALFA^. Samples from control [(C)] and RpH-ILV^3xALFA^–containing sample [transfected/transgene (T)] were loaded as follows: DB (debris), SN (supernatant), FT, and PD (pulldown). The blot was incubated with the ALFA antibody. ALFA pull down of whole lysosome containing samples from transfected S2 cells (**E**) results in ALFA detection in the DB, the SN, and NB, but not in the elution (PD). After the destruction of all organelles by RIPA lysis, (**F**) RpH-ILV^3xALFA^ was detected in the pull-down fraction. Immunopurification of intact RpH-ILV^3xALFA^–containing lysosomes by mechanical lysis (**G**) and RpH-ILV^3xALFA^ after membrane lysis using RIPA (**H**) from transgenic third instar larvae confirmed this observation.

We expressed RpH-ILV^3xALFA^ in *Drosophila* S2 cells by transiently transfecting them with pActSTABLE-RpH-ILV^3xALFA^. Similar to our observations using mammalian cells, RpH-ILV mediated mCherry fluorescence was detected in small punctae, only occasionally colocalizing with pHluorin signal. After the inhibition of lysosomal acidification by treatment with 5 nM BafA1, the pHluorin signal colocalizes almost completely with the mCherry punctae. This suggests a correct localization of RpH-ILV^3xALFA^ in functional lysosomes with a low pH in untreated cells (Fig. 7A). In S2 cells, RpH-ILV was only visible in punctate structures in the mCherry channel. After BafA1 treatment, pHluorin signal was also detected (Fig. 7B), indicating the presence of an active lysosomal v-ATPase.

### RpH-ILV^3xALFA^ is trafficked to lysosomes in transgenic *Drosophila*

The expression of the RpH-ILV construct in transgenic *Drosophila* was carried out by the galactose-responsive transcription factor 4/upstream activating sequence (Gal4/UAS) system using *daugtherless*-Gal4 as driver line (*35*). The localization of the RpH-ILV sensor was verified by coimmunostaining of adipocytes in the fat body of dissected transgenic third instar larvae fixed with 4% formaldehyde (Fig. 7C). As a lysosomal specific marker, ADP-ribosylation factor-like protein 8 (Arl8) was used (*36*). Arl8 signal was observed in ring-like structures, while RpH-ILV signal (mCherry fluorescence) localized inside the Arl8-positive ring. A closer examination of these structures using the ImageJ line plot tool reveals a mCherry signal of variable intensity distributed between the Arl8 signal peaks, confirming RpH-ILV localization inside lysosomes (Fig. 7D).

### The expression of RpH-ILV^3xALFA^ in transgenic flies and in *Drosophila* S2 cells is verified by immunoblotting

RpH-ILV^3xALFA^ with a molecular weight of 89.5 kDa was detected in whole-cell lysates of both third instar larvae and transfected S2 cells using an anti-ALFA antibody (Fig. 7, E to H). Similar to the expression of RpH-ILV^3xALFA^ in HEK293T cells, RpH-ILV^3xALFA^ was lost during lysosomal purification via ALFA beads from both S2 cells and transgenic flies. Mechanical cell lysis destroyed the cells while keeping cell organelles, such as lysosomes, intact. After immunopurification, RpH-ILV^3xALFA^ was detected in the cell debris (DB), soluble supernatant (SN), and nonbound (NB) fraction. In S2 cell culture experiments, a very small part of the RpH-ILV^3xALFA^ was detected in the pull-down fraction of *Drosophila* larvae (Fig. 7E). Only after solubilization of lysosomes with RIPA buffer, the ALFA-tagged RpH-ILV was stably bound by the ALFA beads and detected in the pull-down fraction (Fig. 7F). The observations were similar for transgenic larvae (Fig. 7, G and H), showing that RpH-ILV behaves similarly in a variety of organisms.

### RpH-ILV-marked lysosomes are sensitive to LLOMe-mediated membrane permeabilization

**Fig. 8.**
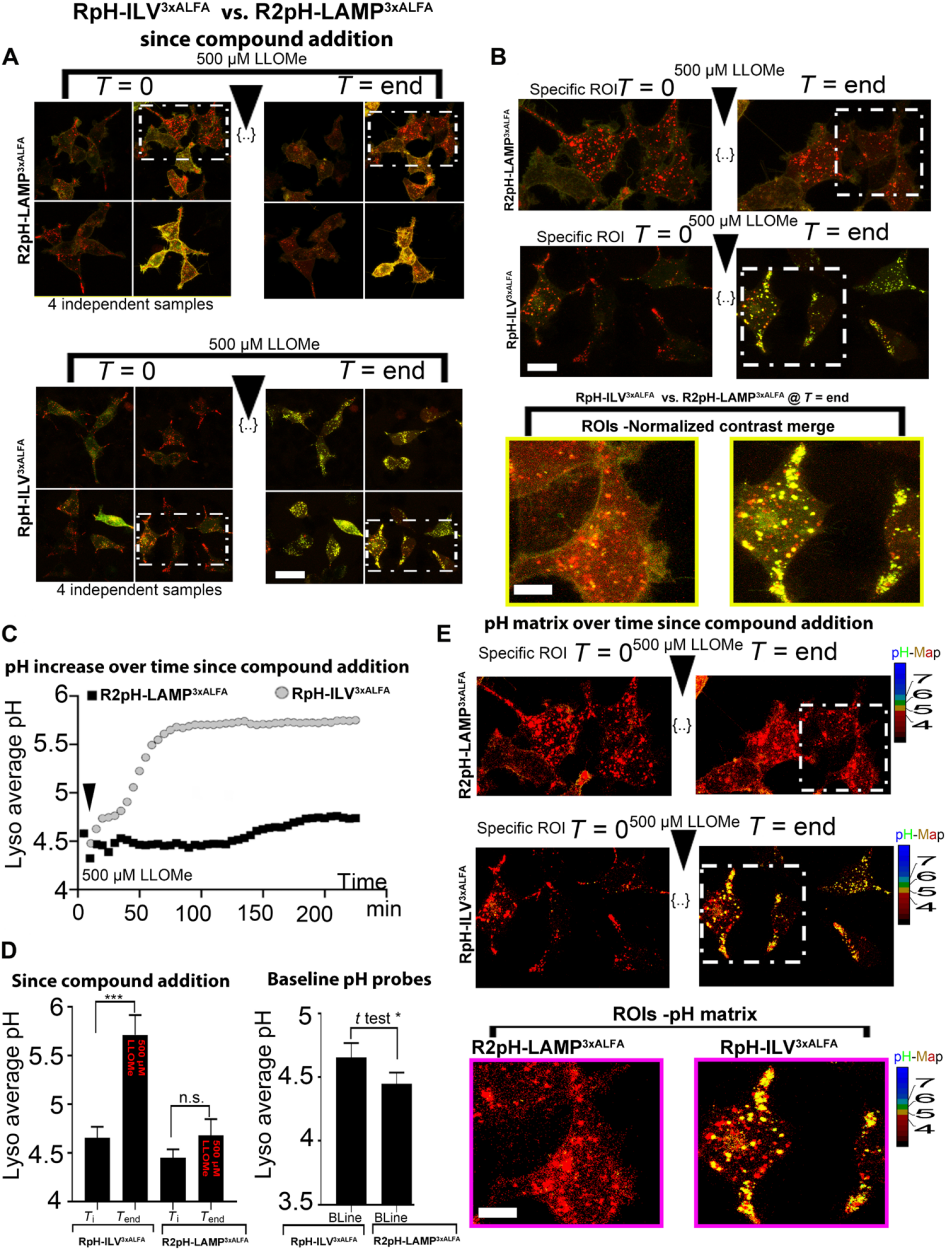
RpH-ILV^3xALFA^ lysosomes are more vulnerable to damage via LLOMe than R2pH-LAMP^3xALFA^. (**A**) Four independent replicates (random fields of view) expressing ratiometric lysosomal sensors and exposed to 500 μM LLOMe-boxed region is shown in (B). Data are acquired in four wells simultaneously. Scale bar, 15 μm. (**B**) ROI expressing lysosomal pH probes. Inset: Single cells after 200-min LLOMe treatment. Scale bars, 10 μm and (ROI) 5 μm. (**C**) Average lysosomal pH per frame in representative movies shown in (B); see also movie S4. (**D**) Lysosomal average pH, before treatment and after treatment with 500 μM LLOMe (six ROIs per probe). (**E**) Same regions of interest as (B), where ratiometric fluorescence has been converted to pH. Scale bar, 5 μm.

We then turned to determine whether RpH-ILV can be used as an index for LLOMe-mediated perforation of the lysosomal limiting membrane. Here, we found that RpH-ILV– and R2pH-LAMP–marked organelles behave differently. We made continuous ratiometric images of labeled lysosomes in four biological replicates in parallel after the addition of 500 μM LLOMe. Whereas lysosomes labeled by R2pH-LAMP respond sluggishly to LLOMe (Fig. 8, A to C and E), maintaining acid pH for at least 100 min after treatment with 500 μM LLOMe, cells expressing RpH-ILV–labeled lysosomes start to lose average lysosomal acidity within about 50 min (Fig. 8C; average lysosomal pH per frame from representative movies in Fig. 8B). Individual RpH-ILV–marked lysosomes within a single cell respond with very variable kinetics (movie S4) and can fluctuate between more neutral to more acid before collapsing and moving permanently to a more alkaline pH. Before treatment (Fig. 8D), RpH-ILV lysosomes appear to be slightly more alkaline than R2pH-LAMP lysosomes (pH 4.65 ± 0.1 versus pH 4.45 ± 0.09, average lysosomal pH in six ROIs per probe, *t* test *P* value = 0.0107) but after LLOMe treatment, average lysosomal pH in RpH-ILV–expressing cells plateaued at pH 5.7 ± 0.1 (average lysosomal pH in six ROIs per probe) (Fig. 8, C and D), with very few RpH-ILV–marked lysosomes becoming any more alkaline than pH 6. In contrast, after LLOMe treatment, R2pH-LAMP lysosomes remained only mildly more alkaline at pH 4.7 ± 0.3. The change in pH before and after LLOMe was significant for the RpH-ILV probe (paired *t* test *P* < 0.0001, average lysosomal pH in six ROIs per probe) and despite trending more alkaline, R2pH-LAMP lysosomes did not significantly change pH after LLOMe treatment.

We then tested if this difference between the two probes extended to other acute lysosomal damaging agents, using the lysosomatropic compound GPN, which is thought to rupture lysosomes (*18*, *37*). We performed experiments in parallel using the v-ATPase inhibitor BafA1 (BafA1) (100 nM) while treating another well with 200 μM GPN. For cells transiently expressing RpH-ILV, both BafA1 and GPN elicited a quick response and unquenching of pHluorin over the 25-min course of the experiment (fig. S5A). To confirm that the effect was related to the RpH-ILV sensor, we also used the same concentration of GPN in Cos7 cells transiently expressing RpH-ILV, which also responded strongly to GPN addition by unquenching (fig. S5B). In the wells transfected with R2pH-LAMP, we see that lysosomes respond to BafA1, but there is minimal effect from GPN treatment, suggesting that R2pH-LAMP expressing cells are resistant to both LLOMe- and GPN-mediated damage.

We then sought to distinguish what kind of damage LLOMe is causing in cells expressing the two probes. Lysosomes move through several stages of damage repair. One of the most commonly used markers of lysosomal damage is recruitment of galectin 3 (Gal3), which recognizes lysosomal rupture by the exposure of β-galactoside sugars of the lysosomal glycocalyx to the cytosol (*17*, *38*). We cloned Gal3 into a haloalkane dehalogenase (Halo)–tagged vector and imaged recruitment of Halo-Gal3 to our probes using an infrared Halo dye. As previously, RpH-ILV cells responded to LLOMe treatment by unquenching, also in the presence of Halo-Gal3, whereas R2pH-LAMP cells remained sluggishly responsive to LLOMe (fig. S6A). Here too, consistent with the lack of changes in pH, suggesting the lack of membrane rupture, RpH-ILV expressing cells show recruitment of Gal3 to lysosomes after LLOMe treatment, whereas R2pH-LAMP–expressing cells do not (fig. S6B and movie S5).

To refine our understanding of this process, we also used an earlier marker of lysosomal damage. Current thinking suggests that earlier lysosomal repair factors are recruited to damaged lysosomes by release of Ca^2+^ from lysosomal stores, which requires much smaller lysosomal ruptures than does the exposure of lysosomal glycocalyx recognized by Gal3. To see if the earlier steps of lysosomal damage are also compromised in R2pH-LAMP–expressing cells, we examined the early lysosomal damage marker, the lipid phosphatidylinositol 4-phosphate (PI4P), which is generated downstream of the Ca^2+^-mediated recruitment of PI4 kinase type II alpha (PI4KIIA) to the damaged lysosomal membrane (*14*). The infrared PI4P probe iRFP-P4M-SidM (*39*) both binds and sequesters PI4P.

Here, we made a notable observation: LLOMe-treated cells expressing both RpH-ILV and R2pH-LAMP showed the production of PI4P on the lysosomal membrane, suggesting that the process of early lysosomal rupture and Ca^2+^ release occurred in lysosomes marked with either probe (fig. S7 and movie S6). However, the presence of a PI4P binding protein in R2pH-LAMP–expressing cells now rendered these lysosomes vulnerable to damage, and these lysosomes now unquench in the presence of LLOMe.

## DISCUSSION

Here, we present a novel ratiometric lysosomal probe, RpH-ILV, which trafficked purely to acid lysosomes, where it was imported to lysosomal luminal structures. The lack of any other structure [such as the plasma membrane pool of LAMP1 we observed with our previous lysosomal sensors with RpH-LAMP (*2*) and R2pH-LAMP (*3*) and its derivatives], allows simpler image processing for live pH calculations and greater flexibility of use.

In addition, the new probe provides a useful complement to the use of R2pH-LAMP probes, as they respond in the same manner to pharmacological stimuli but are trafficked to the lysosome using different adaptor motifs, which will allow the examination of lysosomal phenotypes even when lysosomal adaptor-mediated pathways are compromised.

The RpH-ILV probe appears to be selective for a morphologically distinct set of lysosomes containing ILVs (Fig. 4), likely at the border between rab7-postive late endosomes and their fusion with lysosomes (see immunofluorescence quantification in Fig. 2, where rab7 is revealed as one of the stronger colocalizing factors after CD63 and LAMP1), but these organelles maintain an overall pH (~4.6; Fig. 8D) and the presence of active cathepsin B (Fig. 3) which is consistent with lysosomal function. These lysosomes are positive for markers of the ILV, namely, CD63, but we are unable to detect secreted RpH-ILV in conditioned media (Fig. 4E), suggesting that RpH-ILV is either only colocalized on CD63-positve ILVs, which are not secreted, or RpH-ILV is on a distinct ILV to CD63.

This sensor, despite being localized to a subset of total lysosomes, shows no specific difference to the overall pool of lysosomes marked by LAMP1 overexpression when challenged with pharmacological stressors (see Fig. 6), except for one important feature: Lysosomes expressing RpH-ILV are more vulnerable to LMP induced by the lysosome-permeabilizing agents LLOMe (Fig. 8) and GPN (fig. S5).

Side-by-side comparison also revealed that the average resting pH of RpH-ILV is marginally more alkaline than R2pH-LAMP1–labeled lysosomes (Fig. 8). This may be due to a recently found mechanism mediated by LAMP1: LAMP1 associates with and suppresses (*40*) the Parkinson’s disease–associated lysosomal H^+^-activated H^+^ channel TMEM175 (*6*), further acidifying lysosomes over RpH-ILV–labeled lysosomes.

There are several reasons to believe that the greater vulnerability of RpH-ILV compared to R2pH-LAMP-expressing lysosomes is likely due to the protective functions of LAMP1, rather than our RpH-ILV construct causing lysosomes to be damaged. Several lines of evidence suggest that LAMP1 overexpression could dampen the effect of LMP agents such as LLOMe and GPN: Reduction in LAMP1 and LAMP2 expression sensitizes cancer cells to lysosomal photo-oxidation–mediated permeabilization (*24*), LAMP1 is known to bind to cholesterol (albeit less strongly than LAMP2) and to accept cholesterol from the luminal cholesterol scavenging protein Niemann-Pick intracellular cholesterol transporter 2 (*41*), where cholesterol is then found on the lysosomal limiting membrane during export. Given the importance of cholesterol accumulation at the lysosome in repairing or mitigating LMP (*14*, *42*–*44*), LAMP1 overexpression may make lysosomes more resistant to damage or, equally, more easily repaired. Our studies show that LAMP1-expressing lysosomes lose their resistance to LLOMe-mediated permeabilization in the presence of proteins that sequester the lipid PI4P (fig. S7 and movie S6), which suggests that the ability of R2pH-LAMP–expressing lysosomes to resist LLOMe is dependent on the availability of PI4P.

An interesting feature of LLOMe-induced LMP in RpH-ILV–expressing cells (movie S4) is that (i) individual lysosomes have different pH trajectories, sometimes collapsing quite rapidly and other ones fluctuating before collapsing; (ii) lysosomes, which are beginning to alkalinize, will sort and appear to fuse with other neighboring lysosomes; and (iii) the overall pH of damaged lysosomes once there is a plateau in the LLOMe treatment (approximately 1.5 hours; Fig. 8) is not the pH of the cytosol but closer to neutral (pH 6). Even R2pH-LAMP–expressing lysosomes respond partially to LLOMe and tend to slightly more alkaline over the course of this treatment, although the difference between unstimulated and LLOMe-treated RpH-LAMP–expressing lysosomes was statistically insignificant.

Of interest, RpH-ILV is based on the lysosomal resident protein, TMEM192, which has been labeled previously with acid-sensing probes to investigate lysosomal damage repair. However, in those configurations, the pH sensor is fused to the cytosolic face of full-length TMEM192, which remains on the lysosomal limiting membrane, leading to a sensor that detects engulfment in autophagosomes such that the probe initially reports cytosolic pH, and only becomes acid as the damaged lysosomes are incorporated inside acid autolysosomes (*45*, *46*).

While research is still uncovering the multiple, likely redundant (*15*) pathways of acute lysosomal repair triggered by LMP, it appears that lysosomal repair mechanisms are engaged sequentially, most likely in line with increasing sizes of the perforations of the lysosomal limiting membrane. For this reason, the ability to make kinetic studies of LMP and its repair will be of great use in understanding how these mechanisms coordinate. There have been several time-resolved studies to determine when factors are recruited to damaged lysosomes. In dextran-loaded lysosomes exposed to peroxide, 14-min exposure causes lysosomal 10 kDa dextran (Stoke’s radius ~ 23.6 Å) to leak to the cytosol, whereas 70 kDa dextran (Stoke’s radius ~ 58 Å) is retained (*47*), suggesting that pores are initially limited in size and grow over time. In studies using 0.25 mM LLOMe, this size progression was also seen, but the pores were smaller, only allowing the exit of 4.4 kDa dextran (Stoke’s radius ~ 14 Å) (*48*). A similar phenomenon is observed in the profile of lysosomal hydrolases released to the cytosol over time (*49*), which favors smaller proteins first over larger ones, indicating progressive widening of damage-induced pores.

The progression of damage appears to cue different elements of the damage response: The initial proteins recruited by LLOMe-induced LMP depend on Ca^2+^ release (*14*), which initiates several lysosomal repair pathways. Ca^2+^-triggered recruitment of PI4P-mediated effectors and of ESCRT components occurs within approximately 10 min (*14*), which is also the case for Ca^2+^-activated exposure of sphingomyelin on the lysosomal cytosolic surface (*15*). The later recognition of LMP via Gal3 binding to exposed lysosomal luminal sugars, which follows at around 1 hour after initiation of LMP (*14*), coincides with the loss of lysosomal acidity as measured by LysoTracker (*50*). In our experiments, this coincided with lysosomes reaching a pH of around 6 (Fig. 6), suggesting that there may be residual activity of the lysosomal v-ATPase, even in damaged lysosomes, particularly in the stages before the recruitment of galectin3.

## MATERIALS AND METHODS

### Tissue culture and plasmid transfection

Mammalian cells (please see table S1 for list of cell lines and plasmids) were grown in Dulbecco’s modified Eagle’s medium (DMEM; Gibco, 11995065) supplemented with 1% penicillin/streptomycin (Gibco, 15140122) and 10% fetal bovine serum (Gibco, A5256701), unless otherwise indicated. Cells were passaged regularly to maintain a confluency of between 20 and 80%.

pH measurements were performed in cells transiently transfected using Lipofectamine 2000 (Invitrogen, 11668019) or in clonal cell lines. In the stable inducible line, RpH-ILV^3XALFA^ expression was induced using doxycline hyclate (1 μg/ml; Tokyo Chemical Industry, D4116). Once seeded cells had reached ~60% confluency, they were transfected if required using Lipofectamine 2000. Opti-MEM (Gibco, 31985062) reduced serum medium was used as transfection media.

### Immunofluorescence

Cells were seeded onto glass coverslips and transfected 24 hours later with the construct of interest using the Lipofectamine 2000 (Invitrogen, 11668019) as per the manufacturer’s guidelines. Twenty-four hours following transfection, cells were washed once with phosphate-buffered saline (PBS) and fixed with 4% (w/v) PFA (Polysciences, 04018-1) for 30 min at room temperature. Samples were then permeabilised with either 0.1% Triton X-100 or 0.001% saponin in PBS for 1 hour at room temperature and blocked in 5% (w/v) fatty acid–free bovine serum albumin (BSA)/PBS (Sigma-Aldrich, A7030) for 1 hour at room temperature. Following one wash with PBS, cover slips were incubated with primary antibodies in 5% (w/v) fatty acid–free BSA/PBS overnight at 4°C. Please see table S2 for a list of antibodies and concentrations used. Cells were then washed once with PBS and incubated with secondary antibodies for 1 hour at room temperature in 5% (w/v) fatty acid–free BSA/PBS. Samples were then washed once with PBS and mounted on to SuperFrost Microscope Slides (VWR, 631-0114) with FluorSave Reagent (Millipore, 345789), overnight at room temperature. Samples were then stored at 4°C if not immediately used for imaging.

### Live-cell imaging

Cells (other than neurons, see separate method; please see table S1 for cell lines and plasmids used) were seeded in four-well 35-mm glass bottom CellView imaging dishes (Greiner Bio-One, 627870) approximately 48 hours before imaging and transfected with constructs of interest approximately 24 hours before using Lipofectamine 2000. Transfected Halo-Gal3 was imaged using LIVE RED Halo dye (Abberior, LV-0147), treating the cells 2 hours before imaging following the manufacturer’s instructions.

Images were taken using a Zeiss LSM800 with Airyscan confocal laser scanning microscope with the Zeiss Plan Apochromat 63×/1.40 oil Ph3 8/0.17 microscope objective. During imaging, a microscope stage was used that maintained cells at 37°C and 5% CO_2_.

### Live imaging of lysosomal activity probes

HEK293T cells inducibly expressing lentiviral RpH-ILV^3xALFA^ were seeded onto glass-bottom 35-mm imaging dishes and induced with doxycline hyclate (1 μg/ml; Tokyo Chemical Industry, D4116). Twenty-four hours after induction, cells were loaded with Alexa Fluor 647–dextran (20 μg/ml; Thermo Fisher Scientific, D22914) for 16 hours at 37°C under 5% CO_2_. Cells were subsequently chased for 3 hours at 37°C under 5% CO_2_ before live-cell imaging. For LysoTracker, imaging cells plated as above were treated with 50 nM LysoTracker DND-22 Blue (Invitrogen, L7525) for 1 hour at 37°C under 5% CO_2_. Medium was then changed immediately before live imaging. For cathepsin B activity measurements, cells were loaded overnight with CATB FAST 680 (2 μM; PerkinElmer, NEV11112) at 37°C under 5% CO_2_ before live imaging.

### Neuronal preparation and imaging

Dissociated cortical neurons were obtained from embryonic day 18 C57BL/6 mouse embryos (with thanks to N. Darling, University of Liverpool for providing tissues), following previously described protocols (*51*). The tissue was mechanically dispersed and seeded into eight-well chamber culture dishes pretreated with poly-l-lysine (0.1 mg/ml; Sigma-Aldrich, Germany). Neurons were cultured in Neurobasal A medium (Invitrogen) supplemented with 2% B-27 (Invitrogen) and 0.5% fetal calf serum (PAN Biotech). Cultures were maintained for 15 days. For neuronal transfection, pCAGS-RpH-ILV^3xALFA^ was purified using the EndoFree Maxi Kit (QIAGEN), and cultures were transfected at 7 DIV with Lipofectamine 3000 (Thermo Fisher Scientific, L3000008), following the manufacturer’s instructions, and imaged at 12 to 15 DIV.

### Immunostaining of *Drosophila* larvae

For immunostaining, third-instar larvae were pinned onto Sylgard 184 silicone elastomer plates filled with PBS buffer [PBS; 137 mM NaCl, 2.7 mM KCl, 8 mM Na_2_HPO_4_, and 2 mM KH_2_PO_4_, (pH 7.4)] dissected from the ventral side, fixed with 4% formaldehyde in PBS for 1 hour at room temperature, and washed with BBT [0.1% BSA and 0.1% Tween 20 in PBS) (3× for 15 min). Permeabilization with 1% Triton X-100 in PBS for 1 hour at room temperature and incubation with blocking solution (1% BSA and 0.1% Tween 20 in PBS) for 1 hour at room temperature were followed by incubation with primary antibodies in BBT at 10°C overnight. The primary antibody used was rabbit anti-Arl8 (1:100; Developmental Studies Hybridoma Bank, catalog no. Arl8, RRID: AB_2618258). After washing with BBT (4× for 15 min), the specimens were incubated with secondary antibodies and 1:200 4′,6-diamidino-2-phenylindole in BBT for 2 hours at room temperature. Secondary antibody used was anti–rabbit–Alexa Fluor 633 (1:200; Invitrogen, catalog no. A-21072, RRID: AB_2535733. Samples were then washed with BBT (4× for 15 min) and embedded in Fluoromount-G mounting medium (Thermo Fisher Scientific, Waltham, MA, USA) and an LSM800 (Zeiss) with a 40× oil objective was used for confocal imaging. ImageJ/Fiji was used for image processing (*52*).

### ALFA immunoprecipitation

HEK293T cells with and without the RpH-ILV^3xALFA^ were seeded in 10-cm dishes (in triplicates) with DMEM and 10% FBS, and the construct was induced by the addition of doxycycline 24 hours before harvest. On the day of immunoprecipitation, the growth medium was completely removed and washed carefully with ice-cold PBS. To the cells, 1 ml of lysis buffer [composed of 50 mM sucrose, 20 mM Hepes, 50 mM KCl, 5 mM MgCl_2_, 1 mM EGTA, 2.5 mM adenosine 5′-triphosphate (ATP), and 1× protease inhibitor cocktail] was added and frozen at −80°C for 5 min. The frozen cells were scraped and transferred directly into a dounce homogenizer for cell lysis. The whole-cell lysate was centrifuged at 435*g* for 10 min to pellet the DB, and the supernatant was further spun down at 18,000*g* for 15 min to pellet all the small organelles. This organellar pellet was resuspended in 0.5 ml of pull-down buffer (composed of PBS with 50 mM sucrose, 2.5 mM ATP, 0.5% BSA, and 1× protease inhibitor cocktail) and incubated with 20 μl of magnetic-ALFA Selector ST beads (NanoTag Biotechnologies) for 2 hours at 4°C with 10-rpm rotation. The pulldown samples were placed on the DynaMag stand to magnetically separate the beads and remove the rest of the supernatant. The isolated ALFA beads were washed with 0.5 ml of PBS, and the remaining beads attached to the ALFA tag of the RpH-ILV^3xALFA^ on the lysosomes were either visualized on the Western blot or further processed with the iST PreOmics Sample Preparation Kit for proteomics. In brief, the beads were boiled with the Lyse buffer to denature the proteins and were digested with trypsin/LysC, and the resulting peptides were purified, dried, and resuspended in LC-LOAD from the kit.

For the mass spectrometric analysis via the label-free quantification approach, samples were eluted from a PepMap C18 easy spray column (Thermo Fisher Scientific) with a linear gradient of acetonitrile from 10 to 35% in H_2_O with 0.1% formic acid for 120 min at a constant flow rate of 300 nl/min. The resulting MS and MS/MS spectra were analyzed using MaxQuant and visualized using Perseus.

### *Drosophila* stocks and fly husbandry

The following *Drosophila* lines were used in this work. The control strain white^1118^ (RRID: BDSC_5905) and the driver line *daughterless*-Gal4 (*da*-Gal4, BL95282, RRID: BDSC_55850) were obtained from the Bloomington Drosophila Stock Center (BDSC; Bloomington, IL, USA). A commercial service was used to establish the transgenic UAS line UAS-RpH-ILV^3xALFA^ (BestGene Inc., CA, USA) with the expression plasmid pUASTattB-RpH-ILV. The plasmid was generated by cloning the RpH-ILV^3xALFA^ coding sequence into pUAST-attB (DGRC, stock 1419, RRID: DGRC_1419) and injected into the fly stock RRID: BDSC_24749 on the third chromosome. UAS-RpH-ILV^3xALFA^ was recombined with *da*-Gal4 to obtain the *da*>RpH-ILV^3xALFA^ fly line (*da*-Gal4/CyO; UAS-RpH-ILV^3xALFA^/TM6B) stably expressing RpH-ILV^3xALFA^. Flies were propagated on standard cornmeal agar and raised at room temperature.

### *Drosophila* S2 cell culture and plasmid transfection

S2 cells (RRID: CVCL_Z232) were cultured in Schneider’s *Drosophila* medium (PAN Biotech, catalog no. P04-91500) supplemented with 10% FBS at 30°C as described previously (*53*). The TransIT-Insect Transfection Reagent (Mirus, catalog no. MIR 6100) was used according to the manufacturer’s instructions for transiently transfecting S2 cells with plasmid DNA.

### LLOMe kinetic assay

Cells were seeded in four-well 35-mm glass-bottom CellView imaging dishes (Greiner Bio-One, 627870) approximately 48 hours before imaging. Cells that required transfection with respective plasmids were transfected with the Lipofectamine 2000 reagent 24 hours before imaging. Four-well multi-point imaging was obtained using a Zeiss LSM800 with Airyscan confocal laser scanning microscope with the Zeiss Plan Apochromat 63×/1.40 Oil Ph3 8/0.17 microscope objective. Cells were imaged using the Z-stack function, with LLOMe at a final concentration of 250 or 500 μM being added to each well. LLOMe was prediluted with warm culture medium before the addition to cells. During imaging, a microscope stage was used, which maintained cells at 37°C and 5% CO_2_.

### Lysosome acidification assay in *Drosophila*

S2 cells were cultured and transfected on coverslips and briefly washed with PBS 72 hours after transfection. Afterward, cells were incubated in 5 nM BafA1 (Sigma-Aldrich, catalog no. B1793-2UG) in PBS for 45 min. Control samples were incubated in PBS only. Afterward, the S2 cells were embedded in Fluoromount-G mounting medium (Thermo Fisher Scientific), and the mCherry and pHluorin signals were imaged using an LSM800 (Zeiss) with a 40× oil objective. ImageJ/Fiji was used for image processing.

Third instar larvae, carrying the RpH-ILV^3xALFA^ sensor, were anesthetized and pinned down onto Sylgard plates filled with PBS buffer and dissected from the ventral side (see above). Without fixation, larvae were then treated with 5 nM BafA1 in PBS for 45 min. Then, the larval tissue was embedded in Fluoromount-G mounting medium and imaged as described above.

### SDS-PAGE electrophoresis and Western blot analysis

#### Preparation of cell lysates

Cells were seeded into 60-mm cell culture dishes and lysed with ice-cold RIPA buffer [150 mM NaCl, 1% Triton X-100, 0.1% SDS, 1% sodium deoxycholate, and 10 mM tris-HCl (pH 7.5); Sigma-Aldrich, R0278] supplemented with Protease Inhibitor Cocktail (Sigma-Aldrich, P8340) and PhosSTOP (Roche, PHOS-RO) in accordance with the manufacturer’s instructions. Lysates were incubated at 4°C for 15 min in head over tail incubation and clarified by centrifugation at 12,000 rcf for 10 min at 4°C. The supernatant was transferred to a new tube. Protein concentration was quantified using the Pierce BCA Protein Assay Kit (Thermo Fisher Scientific, 23225) and protein concentrations were equalized using the same supplemented RIPA buffer used to lyse the cells. 5X Laemlli buffer supplemented with β-mercaptoethanol at one-third the volume of the lysates was then added before lysates were boiled at 95°C for 2 min.

#### Immunoprecipitation from conditioned media

Dishes (15 cm) of HEK293T stably expressing inducible RpH-ILV^3xALFA^ were induced 24 hours before the experiment with doxycycline hyclate (1 μg/ml). Cells were then treated with 200 nM BafA1 for 5 hours to stimulate the release of exosomes. Cell pellets were collected in RIPA buffer plus protease inhibitors (cOmplete, Mini EDTA-free protease inhibitor cocktail; Roche, 11836170001) and turbonuclease (Sigma-Aldrich, T4330) and frozen until further analysis. The supernatant was clarified by centrifugation, treated with protease inhibitors, and split in two to be incubated with 20 μl of RFP-Trap (Proteintech, rta) or ALFA Selector PE (NanoTag Biotechnologies, catalog no. N1510) resin overnight. Samples were washed 3× in PBS and treated with RIPA buffer plus protease inhibitors before the addition of boiling Laemmli buffer for electrophoresis and Western Blot.

#### SDS-PAGE electrophoresis and Western blot analysis

The SDS–polyacrylamide gel electrophoresis (SDS-PAGE) gels were prepared at 10% bis-acrylamide (resolving gel) and 5% bis-acrylamide (stacking gel) using bis-acrylamide (National Diagnostics, EC890), tris-base (pH 6.8) (Fisher Bioreagents, BP152-1), SDS (Sigma-Aldrich, L5750), 10% (w/v) ammonium persulfate (Sigma-Aldrich, L5750), and tetramethyl ethylenediamine (Sigma-Aldrich, T9281). The SDS-PAGE gels were loaded with 30 μg of protein from prepared cell lysates and PageRuler Plus Prestained Protein Ladder (10 to 250 kDa; Thermo Fisher Scientific, 26619). Samples were resolved in 1X running buffer at 130 V until the bromophenol blue had started to leak out of the gels base. Resolved gels were transferred onto 0.45-μm nitrocellulose membranes (Cytiva, 1060003) at 100 V for 90 min in 1X transfer buffer. The transfer was then validated by Ponceau staining. Membranes were then blocked for 1 hour at room temperature in 5% (w/v) milk TBS-T (tris-buffered saline supplemented with 0.1% Tween 20). Primary antibodies were incubated at their respective concentrations overnight at 4°C in the same solution used for blocking. Antibodies were used as follows: mouse anti-CD63 (1:200; Santa Cruz Biotechnology, Sc-5275), biotinylated-sdAB anti-ALFA (1:3000; NanoTag Biotechnologies, N1505), and mouse anti–glyceraldehyde-3-phosphate dehydrogenase (1:1000; Proteintech, 1E6D9).

Membranes were washed three times, 10 min at a time, with TBS-T before being incubated for 1 hour at room temperature with horseradish peroxidase–conjugated secondary antibodies: goat anti–mouse-HRP (1:20,000; Thermo Fisher Scientific, 31430) or streptavidin-HRP (1:30,000; Thermo Fisher Scientific, s911). Membranes were washed again three times with TBS-T, 10 min at a time, before being incubated with the SuperSignal West Dura Extended Duration Substrate (Thermo Fisher Scientific, 34075) prepared in accordance with the manufacturer’s instructions for 5 min. Chemiluminescence was visualized using the Bio-Rad ChemiDoc MP imaging system.

#### Immunoelectron microscopy

Cells were fixed for 2 hours at room temperature in periodate lysine formaldehyde [0.01 M periodate, 0.075 M lysine, and 2% formaldehyde in 0.037 M phosphate buffer (pH 7.4)]. After fixative washout with 1X PBS, cells were incubated with 50 mM glycine and blocked for 30 min in blocking buffer (0.2% bovine serum albumin, 5% goat serum, 50 mM NH_4_Cl, 0.1% saponin, 20 mM PO_4_ buffer, and 150 mM NaCl) at room temperature. PO_4_ buffer was prepared as follows: Two separate stock solutions are prepared—14.2 g of sodium phosphate dibasic in 500 ml of water and 4.8 g of sodium phosphate monobasic in 200 ml of water. To prepare 500 ml of 0.2 M PO_4_ buffer (pH 7.4), monobasic stock solution was added to 400 ml of dibasic stock solution until pH 7.4 is reached.

Samples were sequentially labeled with primary rabbit anti-ALFAtag (NanoTag Biotechnologies, N1580) and Alexa Fluor 647/nanogold-labeled secondary antibodies (Nanoprobes, 7516) in blocking buffer at room temperature. Cells were refixed for 30 min in 1% glutaraldehyde, and the nanogold particles were enlarged with gold enhancement solution (Nanoprobes) according to the manufacturer’s instructions. Cells were post-fixed with OsO_4_ and processed as described for electron microscopy. Images were acquired with a Ceta charge-coupled device camera (FEI, Thermo Fisher Scientific) using Velox 3.6.0 (FEI, Thermo Fisher Scientific) on Talos L120C TEM (FEI, Thermo Fisher Scientific) operating at 120 kV.
