# Supplementary material for: RpH-ILV: Probe for lysosomal pH and acute LLOMe-induced membrane permeabilization in cell lines and Drosophila
Source: Sci Adv. 2025 Jan 3;11(1):eadr7325. doi: 10.1126/sciadv.adr7325 (PMC11698090; doi:10.1126/sciadv.adr7325)
Supplement: Supplementary file 1 — Figs. S1 to S7 Tables S1 and S2 Legends for movies S1 to S6 [file sciadv.adr7325_sm.pdf]

Science Advances
AAAS

Supplementary Materials for

RpH-ILV: Probe for lysosomal pH and acute LLOMe-induced membrane permeabilization in cell lines and *Drosophila*

Izaak J. Cheetham-Wilkinson *et al.*

Corresponding author: Laura E. Swan, laura.swan@liverpool.ac.uk;
Massimiliano Stagi, massimiliano.stagi@liverpool.ac.uk

Sci. Adv. **11**, eadr7325 (2025)
DOI: 10.1126/sciadv.adr7325

The PDF file includes:

Figs. S1 to S7
Tables S1 and S2
Legends for movies S1 to S6
References

Other Supplementary Material for this manuscript includes the following:

Movies S1 to S6

Supplementary Materials

Supplementary Figure 1 (related to Figure 1) Live imaging of a variety of cell culture lines show RpH-ILV^{3xALFA} is trafficked to acidic compartments where pHluorin is quenched. Human dermal fibroblasts, mouse primary lung fibroblasts, HuH7 (human hepatoma), C2BBe1 (subclone of Caco-2 human colorectal cancer), HeLa (human uterine tumour), BHK (baby hamster kidney), MFC17 (subclone of NIH-3T3 mouse fibroblasts).

Supplementary Figure 1

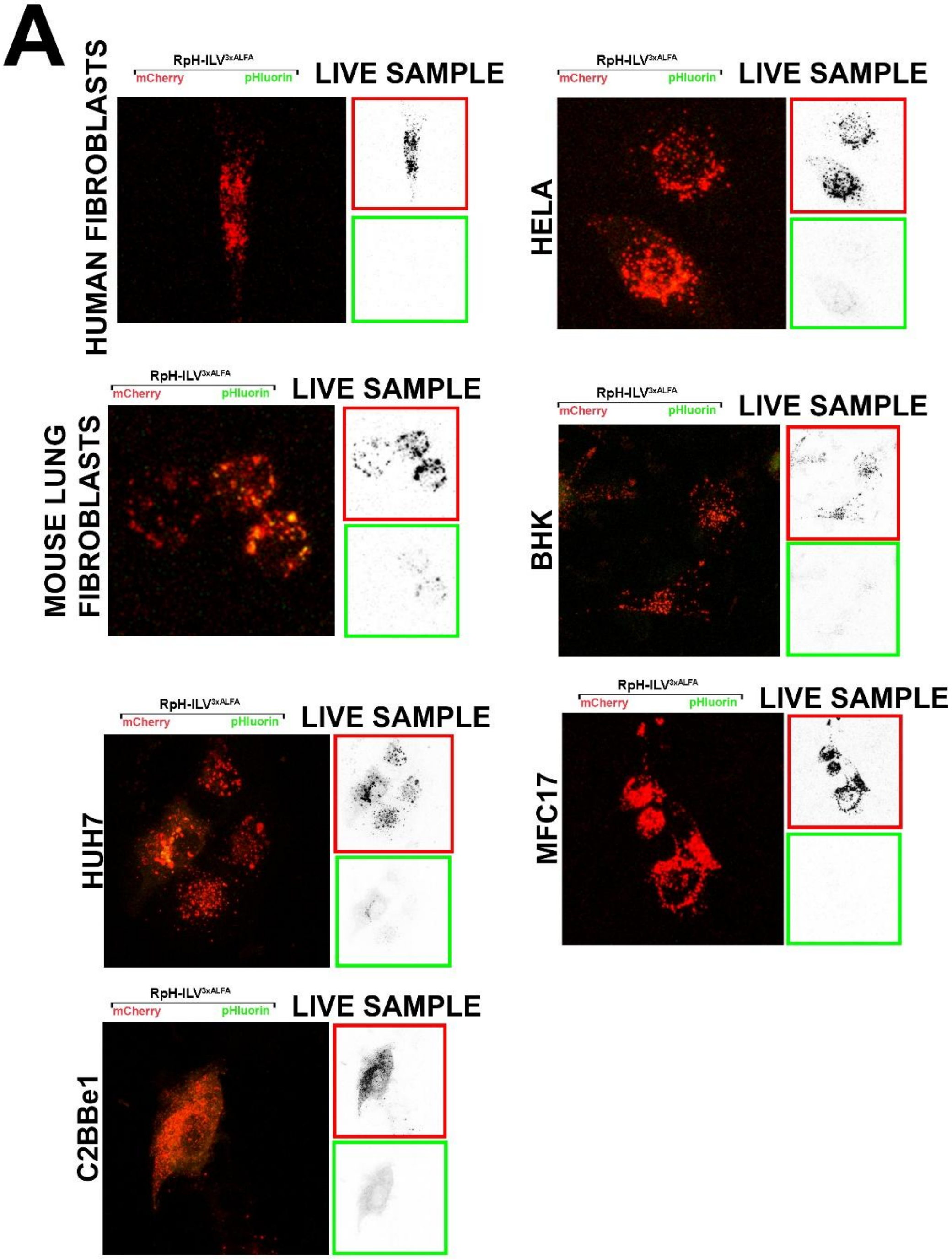

Izaak J Cheetham-Wilkinson et al.

Supplementary Figure 2 (related to Figure 1) Cells transiently expressing RpH-ILV^{3xALFA} respond to inhibition of the lysosomal V-ATPase by treatment with Bafilomycin A1 (100nM) and the pore-forming toxin Nigericin (10μM) by unquenching pHluorin fluorescence. *Far left column*, cells before treatment, *Far right column*, cells after 25mins treatment. Central columns, pH calculations before and after treatment. Refer to **Supplemental Movie 1** for live imaging of Cos7 cells treated as above.

Supplementary Figure 2

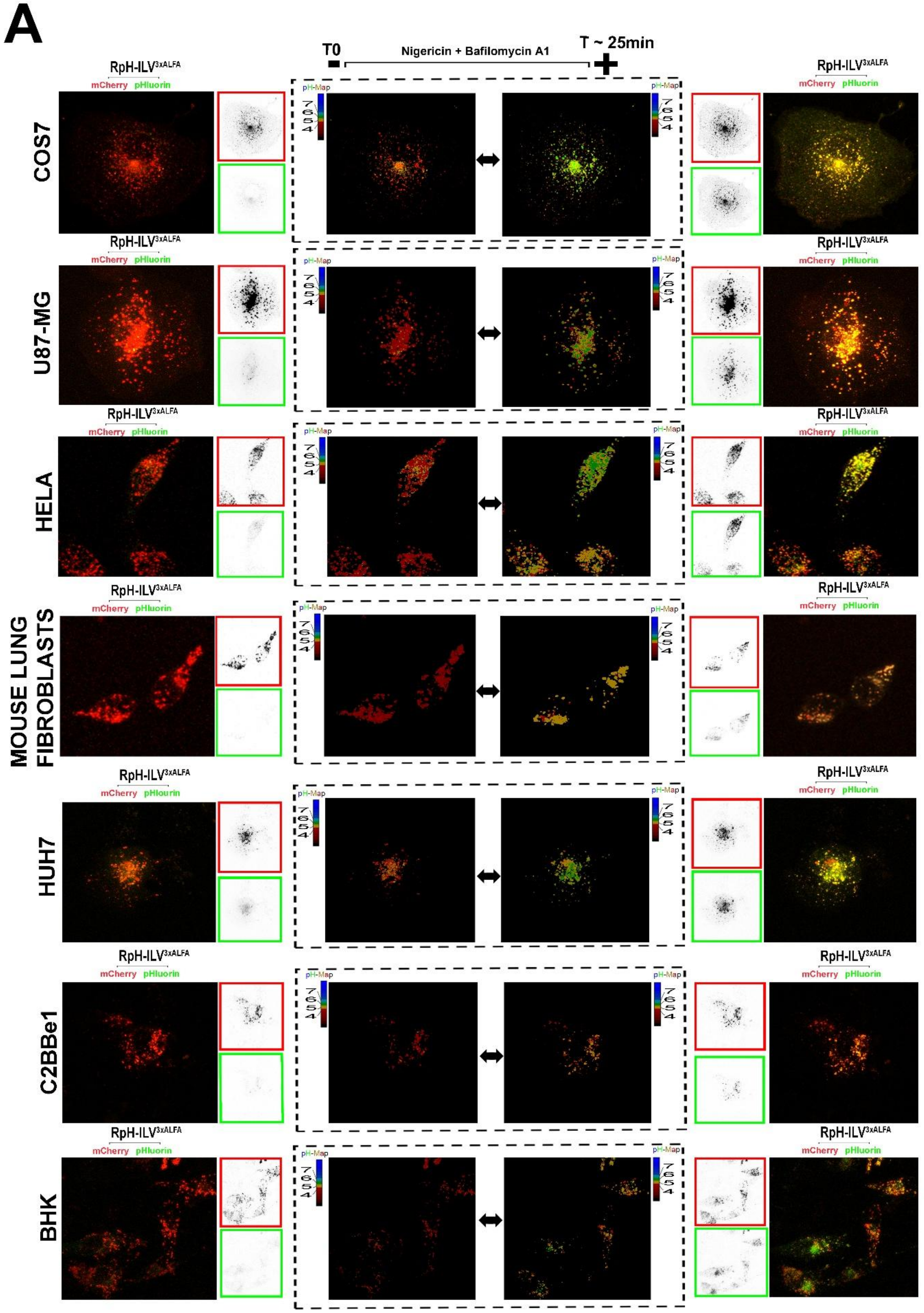

Izaak J Cheetham-Wilkinson et al.

Supplementary Figure 3 (related to Figure 1) Neurons expressing RpH-ILV^{3xALFA} were imaged using time-lapse microscopy at 12 -15*d.i.v*.

A) Live imaging of RpH-ILV^{3xALFA}-expressing neuron showing typical morphology. *scale bar*:10 µm **B**) timelapse imaging of neuron in A (Refer to **Supplemental Movie 3**). pH remains stable over the course of imaging. **C**) Kymograph of region (x-axis representing a 75 µm segment-see dotted yellow line, y-axis 22 minutes), showing the movement of acid lysosomes labelled with RpH-ILV^{3xALFA} in neuronal processes **D**) blue box in C, showing the presence of both immobile and mobile acid compartments labelled with RpH-ILV^{3xALFA}. **E**) Further example of neuronal cell transfected with RpH-ILV^{3xALFA}. **F**) Cell in E treated with Bafilomycin A1 (100nM) and nigericin (10µM) reveals lysosomal pHluorin fluorescence (Refer to **Supplemental Movie 2**).

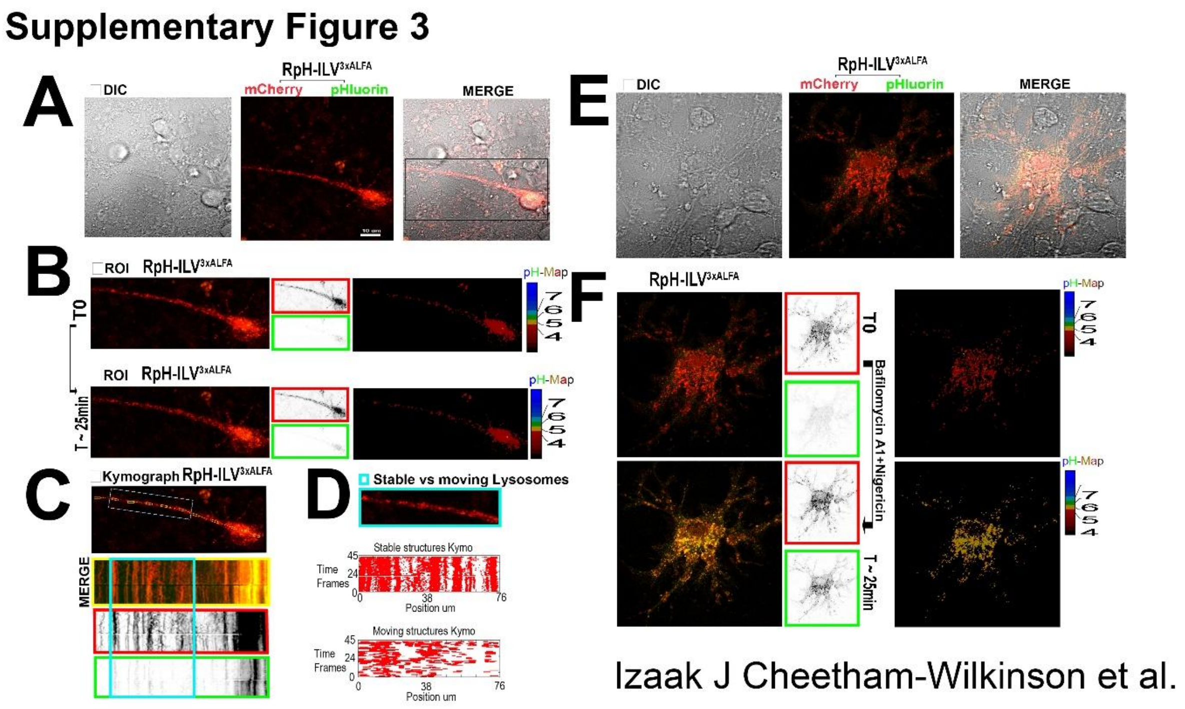

Supplementary Figure 4, related to Figure 2 CD63 immunostaining in **A**) non-neuronal cells and **B**)15 *d.i.v*. primary mouse culture shows RpH-ILV^{3xALFA} colocalizes with CD63 in transfected cells. *Scale Bars*: 10μm in main image, 3μm in boxed ROI.

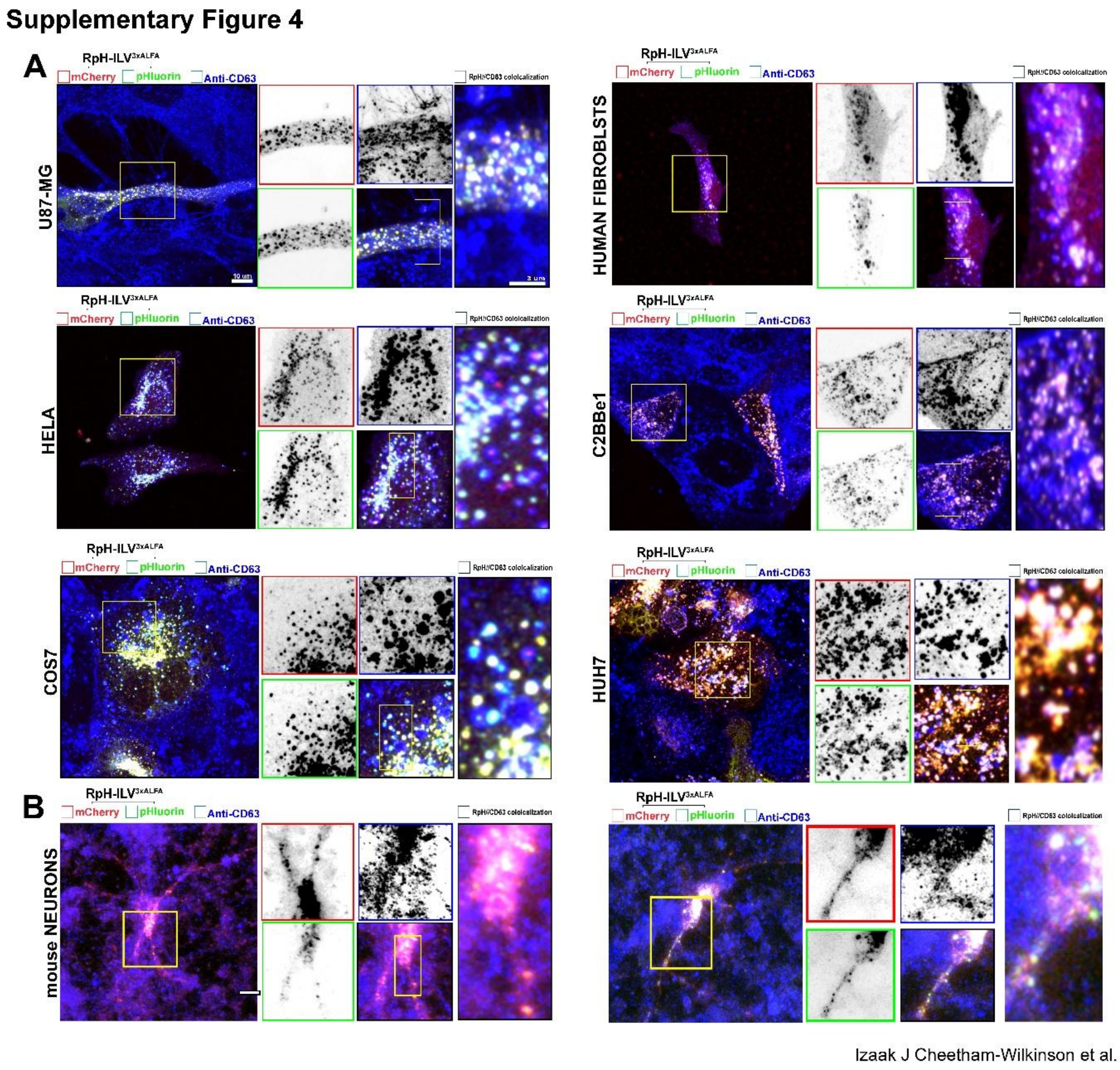

Supplementary Figure 5, related to Figure 8 Response of probes to lysosomal damaging agents. *Far left column*, fluorescence image before treatment, *center left column*, pH calculation before treatment, *center right column*, fluorescence image 25 minutes after treatment, *far right column*, pH calculation 25 minutes after treatment.

A) Treatment of HEK293T cells transiently expressing RpH-ILV^{3xALFA} shows response within 25 minutes to both Bafilomycin A1 (100nM) and to GPN (200 μM)
B) Transfection of RpH-ILV^{3xALFA} in Cos7 cells shows robust response to 200 μM GPN.
C) Transient transfection of R2pH-LAMP^{3xALFA} in HEK293T cells. Cells respond to Bafilomycin A1 treatment, but respond very little to treatment with RpH-ILV^{3xALFA} GPN over the course of 25 minutes.

Supplementary Figure 5

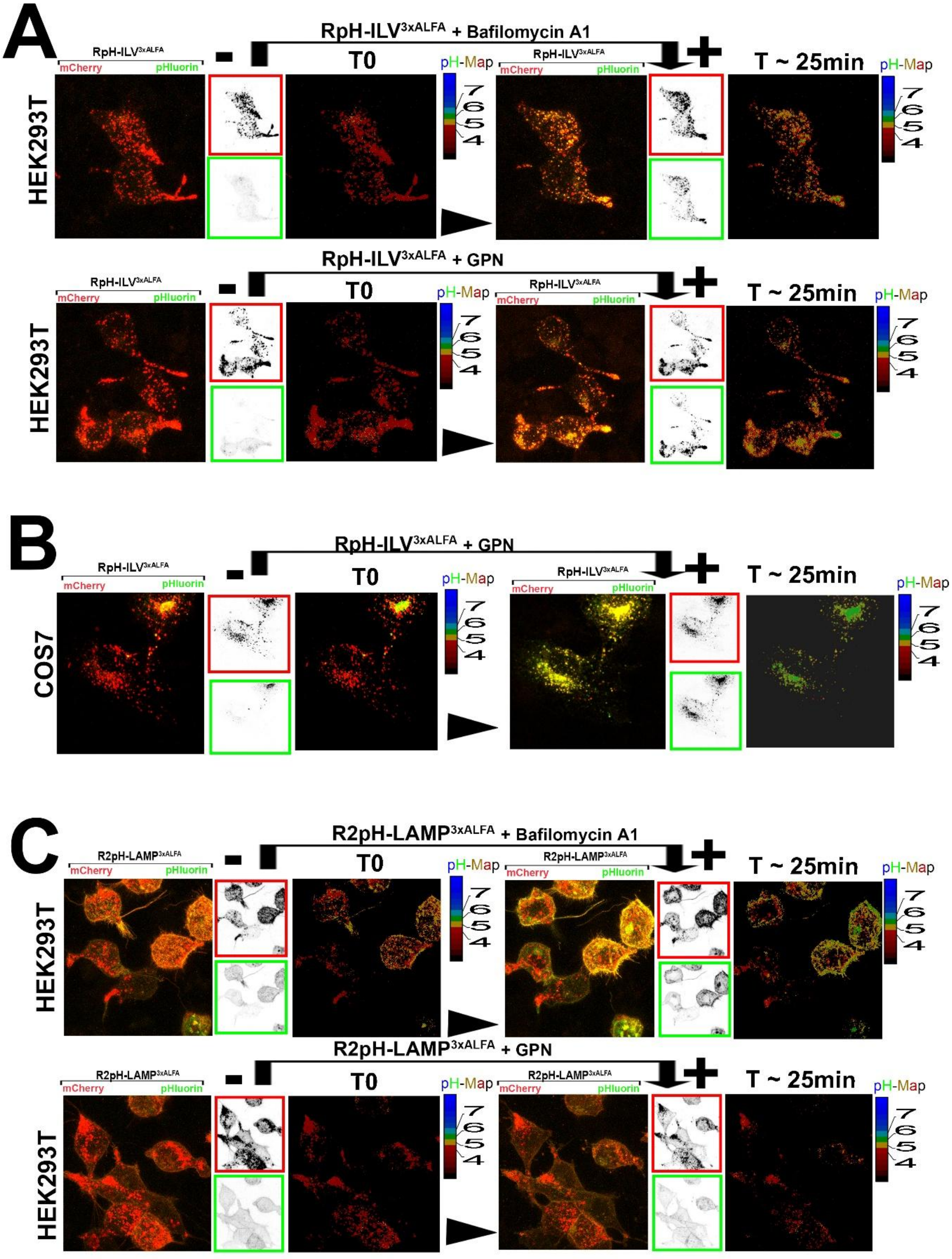

Izaak J Cheetham-Wilkinson et al.

Supplementary Figure 6, related to Figure 8 Co-expression of R2pH-LAMP^{3xALFA} or RpH-ILV^{3xALFA} probes with Halo-tagged Gal3 does not change response to 500µM LLOMe. *Left panel*, HEK293T cells transiently expressing RpH-ILV^{3xALFA} and Halo-Gal3 start to unquench in response to LLOMe-mediated damage within 15-20 minutes, whereas *Right panel*: cells expressing R2pH-LAMP^{3xALFA} and Halo-Gal3 do not respond by unquenching in the presence of LLOMe. *Centre panels*; pH calculations **B**) recruitment of Halo-Gal3 to RpH-ILV^{3xALFA} positive puncta (*left*), but not R2pH-LAMP^{3xALFA} puncta (*right*). Maximum projection of z stack (30 slices), 1 frame every 1.5 minutes. See **Supplementary Movie 5** for live imaging.

Supplementary Figure 6

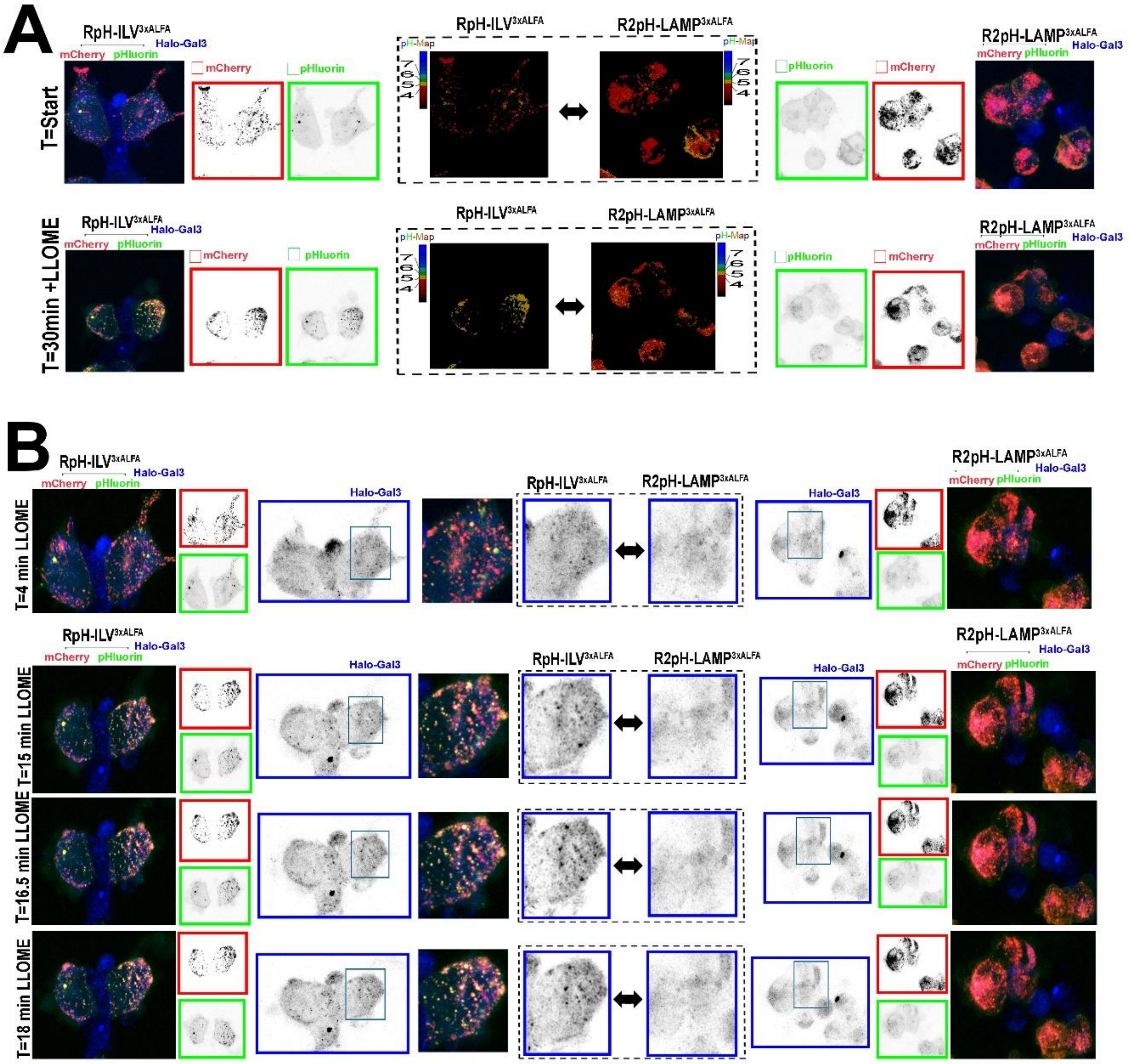

Izaak J Cheetham-Wilkinson et al.

Supplementary Figure 7, related to Figure 8 Both R2pH-LAMP^{3xALFA} and RpH-ILV^{3xALFA} respond to LLOMe treatment by generating PI4P on lysosomes. Cells were transiently transfected with lysosomal pH probes and the PI4P binding protein iRFP-P4M-SidM, then treated with 250μM LLOMe. Wells expressing R2pH-LAMP^{3xALFA} and RpH-ILV^{3xALFA} were treated with LLOMe and imaged simultaneously. Images taken from a single imaging plane, one frame every 12s **A**) RpH-ILV^{3xALFA}/iRFP-P4M-SidM transfected cells before (*left*) and 36 minutes after (*right*) treatment with 250μM LLOMe. Lysosomes become PI4P-postive, and the RpH-ILV^{3xALFA} probe unquenches. S*cale bar*: 10μm **B**) R2pH-LAMP^{3xALFA}/iRFP-P4M-SidM transfected cells exposed to 250μM LLOMe, before (*left*) and 37 mins after (*right*) treatment. In the presence of iRPF-P4M-SidM, R2pH-LAMP^{3xALFA}-expressing cells unquench their lysosomal pHluorin after LLOMe treatment. *Scale bar*: 10μm. See **Supplementary Movie 6.**

Supplementary Figure 7

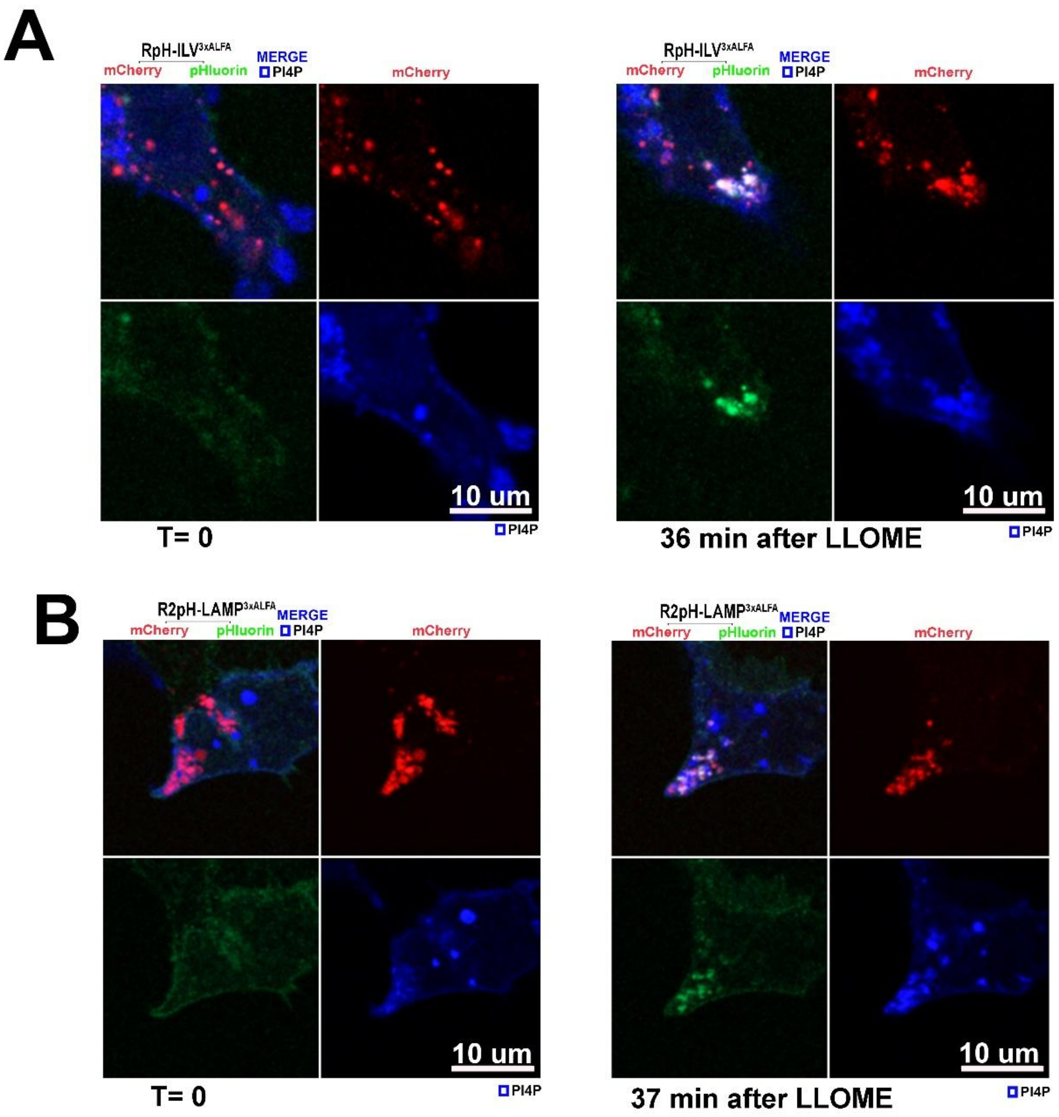

Izaak J Cheetham-Wilkinson et al.

Supplementary Tables:

Supplementary Table 1:
List of cell lines and plasmids used.

Cell line	*Description*	*Origin*
HEK293T	Human embryonic kidney, transformed with SV40 large T antigen. HEK293T (RRID:CVCL_0063)	*Previous work*: (*2*)
HEK293T: CW57-RpH-ILV^{3XALFA}	Stable line inducibly expressing RpH-ILV^{3XALFA} probe	*This work*
HEK293T: CW57-R2pH-LAMP^{3XALFA}	Stable line expressing R2pH-LAMP^{3XALFA} probe	*This work*
Human Fibroblasts	F152-14N-BM human dermal fibroblasts	Prof Volker Straub, Newcastle University Biobank
U-87 MG	Human glioblastoma astrocytoma<br>U-87MG ATCC (RRID:CVCL_0022)	ECACC General Cell Collection (ECACC 89081402)
HeLa	Human uterine adenocarcinoma epithelia cells<br>HeLa (RRID:CVCL_0030)	ATCC (CCL-2)
Mouse Lung fibroblasts	Primary lung fibroblast culture	*This work*
MFC17	Cell line derived from<br>NIIH 3T3 mouse embryonic fibroblasts<br>(17-Cl1;<br>RRID:CVCL_VT75)	Kind gift of Dr Maximilian Erdmann and Dr Edward Emmott, University of Liverpool
HuH7	Human hepatoma cell line (RRID:CVCL_0336)	Kind gift of Dr Maximilian Erdmann and Dr Edward Emmott, University of Liverpool
C2BBe1	Subclone of Caco-2<br>Human colorectal adenocarcinoma<br>(RRID:CVCL_1096)	Kind gift of Dr Maximilian Erdmann and Dr Edward Emmott, University of Liverpool
BHK	Baby Hamster Kidney cells<br>BHK-21 clone 13 (RRID:CVCL_1915)	Kind gift of Dr Maximilian Erdmann and Dr Edward Emmott, University of Liverpool
**Plasmid name**	**Description**	**Obtained from**
pCAGS-R2pH-LAMP^{3xALFA}	Ratiometric sensor with cytosolic 3xALFA affinity tag	*This work*

pCAGS-R2pH-LAMP-FRB	Ratiometric sensor with cytosolic rapamycin-dependent recruitment tag	*This work*
pCAGS-RpH-ILV^{3xALFA}	Ratiometric sensor with ‘cytosolic’ 3xALFA affinity tag	*This work*
pCW57.1-RpH-ILV^{3xALFA}	Stable inducible expression of RpH-ILV^{3xALFA}	*This work*<br>Backbone:<br>CW57.1 pGK-rtTA-2A-puro<br>Addgene:41393, gift of David Root.
pCAGS-RpH-ILV-FRB	Ratiometric sensor with ‘cytosolic’ rapamycin-dependent recruitment tag	*This work*
CFP-FKBT	Recruitable cytosolic CFP	Addgene:20160 (*54*)
LAMP1^{miRFP703}	Infrared fluorescent Lamp1	Addgene:79998 (*55*)
pAcV-STABLE-RpH-ILV^{3xALFA}	Expression of RpH-ILV in *Drosophila S2*	*This work*
pUASTattB-RpH-ILV^{3xALFA}	Expression of RpH-ILV in transgenic flies	*This work*
iRFP-P4M-SidM	Detection and binding of PI4P	Addgene:51470 (*39*)
Halo-Gal3	Detection of lysosomal rupture and exposure of glycocalyx	*This work*<br>Subcloned from pEGFP-Gal3; Addgene:73080 (*49*)

Supplementary Table2:
List of antibodies used for immunofluorescence studies.

Immunocytochemistry:

**Antibody**	**Cat. No.**	**Dilution**	**Obtained from**
nanobody αALFA	N1502	1:500	Nanotag Biotechnologies
mouse αCD63	Sc-5275	1:100	Santa Cruz
mouse αLAMP1	555798	1:100	BD Pharmingen
rabbit αRab7	ab137029	1:200	Abcam
mouse αLAMP2	ab25631	1:100	Abcam
mouse αVPS35	sc-37472	1:100	Santa Cruz
mouse αLBPA	MABT837	1:250	Sigma-Aldrich
rabbit αTGN46	13573-1-AP	1:200	Proteintech
mouse αM6PR	ab2733	1:100	Abcam
rabbit αEEA1	3288S	1:200	Cell Signalling Technology
rabbit αGM130	ab52649	1:200	Abcam
rabbit αPEX14	10594-1-AP	1:500	Proteintech
rabbit αRab5a	11947-1-AP	1:100	Proteintech
mouse αCytC	612302	1:1000	Biolegend
rabbit αArl8 (fly)	Arl8	1:100	Developmental Studies Hybridoma Bank

Supplementary Movies

Supplementary Movie 1: related to Figure 1, RpH-ILV^{3xALFA}-expressing Cos7 cell responding to BafilomycinA1 (100nM) plus Nigericin (10μM) treatment. Maximum projection of 8 Z-plane stack, Frame rate 0.1Hz, total movie 29.5 minutes.

Supplementary Movie 2: related to Figure 1, Neuron imaged in Supplementary Figure 3E, F. RpH-ILV^{3xALFA}-transfected primary mouse neuron responding to BafilomycinA1 (100nM) and Nigericin (10μM) treatment. Images from a single Z plane, 1 frame every 30 seconds, total movie 14.5 minutes.

Supplementary Movie 3: related to Figure 1, Neuron imaged in Supplementary Figure 3A-D. Images were acquired at a rate of one Z compressed frame (20 images per stack) every 30 seconds over a total duration of 22 minutes. *scale bar* :10μm. *Left column*, merged image and separated mCherry and pHluorin channels. *Bottom panel*, pH calculation. *Right column, top to bottom*: raw imaging data for kymograph, thresholded image and image segregated to individual particles, kymograph of stationary and moving particles. Kymograph x-axis: segment length (75 μm), y-axis: time (22 minutes).

Supplementary Movie 4: related to Figure 8. R2pH-LAMP does not respond significantly to LLOMe treatment, whereas RpH-ILV unquenches in response to LLOMe. 4 independent wells transfected with lysosomal probes, treated with 500 μM and acquired in parallel. Maximum projections of Z-stacks, 1 frame every 5 minutes. *Upper panels* R2pH-LAMP, Lower panels, RpH-ILV. Boxed region, pH calculation in ROI indicated.

Supplementary Movie 5: related to Figure 8. Halo-Gal3 is not recruited to R2pH-LAMP expressing lysosomes treated with 500μM LLOMe. RpH-ILV (*upper panel*) and R2pH-LAMP (*lower panel*) co-transfected with Halo-Gal3, revealed by Abberior LIVE RED Halo ligand, and treated with LLOMe. Maximum projection of Z stacks, one frame every 1.5mins.

Supplementary Movie 6: related to Figure 8. The early lysosomal damage marker, PI4P is present on LLOMe treated lysosomes using both RpH-ILV and R2pH-LAMP probes. *Scale bar* 10μm. Single imaging plane, 1 frame every 12s. *Upper panel*: RpH-ILV marked lysosomes recruit PI4P after 250μM LLOMe treatment *Lower panel*: R2pH-LAMP marked lysosomes also recruit PI4P after 250μM LLOMe treatment, but unlike previous treatments, R2pH-LAMP pHluorin unquenches in the presence of a PI4P binding protein.

REFERENCES AND NOTES

1. R. E. Lawrence, R. Zoncu, The lysosome as a cellular centre for signalling, metabolism and quality control. *Nat. Cell. Biol.* **21**, 133–142 (2019).

2. A. H. Ponsford, T. A. Ryan, A. Raimondi, E. Cocucci, S. A. Wycislo, F. Frohlich, L. E. Swan, M. Stagi, Live imaging of intra-lysosome pH in cell lines and primary neuronal culture using a novel genetically encoded biosensor. *Autophagy* **17**, 1500–1518 (2021).

3. J. A. Kraut, I. J. Cheetham-Wilkinson, L. E. Swan, M. Stagi, I. Kurtz, Impact of various buffers and weak bases on lysosomal and intracellular pH: Implications for infectivity of SARS-CoV-2. *FASEB Bioadv.* **5**, 149–155 (2023).

4. R. J. Mulligan, M. M. Magaj, L. Digilio, S. Redemann, C. C. Yap, B. Winckler, Collapse of late endosomal pH elicits a rapid Rab7 response via V-ATPase and RILP. *J. Cell Sci.* **137**, jcs261765 (2024).

5. Z. A. Klein, H. Takahashi, M. Ma, M. Stagi, M. Zhou, T. T. Lam, S. M. Strittmatter, Loss of TMEM106B ameliorates lysosomal and frontotemporal dementia-related phenotypes in progranulin-deficient mice. *Neuron* **95**, 281–296.e6 (2017).

6. M. Hu, P. Li, C. Wang, X. Feng, Q. Geng, W. Chen, M. Marthi, W. Zhang, C. Gao, W. Reid, J. Swanson, W. Du, R. I. Hume, H. Xu, Parkinson's disease-risk protein TMEM175 is a proton-activated proton channel in lysosomes. *Cell* **185**, 2292–2308.e20 (2022).

7. I. Sava, L. J. Davis, S. R. Gray, N. A. Bright, J. P. Luzio, Reversible assembly and disassembly of V-ATPase during the lysosome regeneration cycle. *Mol. Biol. Cell* **35**, ar63 (2024).

8. J. A. Mindell, Lysosomal acidification mechanisms. *Annu. Rev. Physiol.* **74**, 69–86 (2012).

9. L. Fernandez-Mosquera, K. F. Yambire, R. Couto, L. Pereyra, K. Pabis, A. H. Ponsford, C. V. Diogo, M. Stagi, I. Milosevic, N. Raimundo, Mitochondrial respiratory chain deficiency inhibits lysosomal hydrolysis. *Autophagy* **15**, 1572–1591 (2019).

10. K. M. Stepien, F. Roncaroli, N. Turton, C. J. Hendriksz, M. Roberts, R. A. Heaton, I. Hargreaves, Mechanisms of mitochondrial dysfunction in lysosomal storage disorders: A review. *J. Clin. Med.* **9**, 2596 (2020).

11. J. Jia, Y. P. Abudu, A. Claude-Taupin, Y. Gu, S. Kumar, S. W. Choi, R. Peters, M. H. Mudd, L. Allers, M. Salemi, B. Phinney, T. Johansen, V. Deretic, Galectins control mTOR in response to endomembrane damage. *Mol. Cell.* **70**, 120–135.e8 (2018).

12. K. F. Yambire, L. Fernandez-Mosquera, R. Steinfeld, C. Muhle, E. Ikonen, I. Milosevic, N. Raimundo, Mitochondrial biogenesis is transcriptionally repressed in lysosomal lipid storage diseases. *eLife* **8**, e39598 (2019).

13. R. Desai, A. E. Frazier, R. Durigon, H. Patel, A. W. Jones, I. Dalla Rosa, N. J. Lake, A. G. Compton, H. S. Mountford, E. J. Tucker, A. L. R. Mitchell, D. Jackson, A. Sesay, M. Di Re, L. P. van den Heuvel, D. Burke, D. Francis, S. Lunke, G. McGillivray, S. Mandelstam, F. Mochel, B. Keren, C. Jardel, A. M. Turner, P. Ian Andrews, J. Smeitink, J. N. Spelbrink, S. J. Heales, M. Kohda, A. Ohtake, K. Murayama, Y. Okazaki, A. Lombes, I. J. Holt, D. R. Thorburn, A. Spinazzola, ATAD3 gene cluster deletions cause cerebellar dysfunction associated with altered mitochondrial DNA and cholesterol metabolism. *Brain* **140**, 1595–1610 (2017).

14. J. X. Tan, T. Finkel, A phosphoinositide signalling pathway mediates rapid lysosomal repair. *Nature* **609**, 815–821 (2022).

15. P. Niekamp, F. Scharte, T. Sokoya, L. Vittadello, Y. Kim, Y. Deng, E. Sudhoff, A. Hilderink, M. Imlau, C. J. Clarke, M. Hensel, C. G. Burd, J. C. M. Holthuis, Ca^{2+}-activated sphingomyelin scrambling and turnover mediate ESCRT-independent lysosomal repair. *Nat. Commun.* **13**, 1875 (2022).

16. N. Kaur, L. R. de la Ballina, H. S. Haukaas, M. L. Torgersen, M. Radulovic, M. J. Munson, A. Sabirsh, H. Stenmark, A. Simonsen, S. R. Carlsson, A. H. Lystad, TECPR1 is activated by damage-induced sphingomyelin exposure to mediate noncanonical autophagy. *EMBO J.* **42**, e113105 (2023).

17. S. Aits, J. Kricker, B. Liu, A. M. Ellegaard, S. Hamalisto, S. Tvingsholm, E. Corcelle-Termeau, S. Hogh, T. Farkas, A. Holm Jonassen, I. Gromova, M. Mortensen, M. Jaattela, Sensitive detection of lysosomal membrane permeabilization by lysosomal galectin puncta assay. *Autophagy* **11**, 1408–1424 (2015).

18. M. L. Skowyra, P. H. Schlesinger, T. V. Naismith, P. I. Hanson, Triggered recruitment of ESCRT machinery promotes endolysosomal repair. *Science* **360**, eaar5078 (2018).

19. J. H. Kluss, A. Beilina, C. D. Williamson, P. A. Lewis, M. R. Cookson, L. Bonet-Ponce, Lysosomal positioning regulates Rab10 phosphorylation at LRRK2$^+$ lysosomes. *Proc. Natl. Acad. Sci. U.S.A.* **119**, e2205492119 (2022).

20. L. Bonet-Ponce, A. Beilina, C. D. Williamson, E. Lindberg, J. H. Kluss, S. Saez-Atienzar, N. Landeck, R. Kumaran, A. Mamais, C. K. E. Bleck, Y. Li, M. R. Cookson, LRRK2 mediates tubulation and vesicle sorting from lysosomes. *Sci. Adv.* **6**, eabb2454 (2020).

21. T. Eguchi, T. Kuwahara, M. Sakurai, T. Komori, T. Fujimoto, G. Ito, S. I. Yoshimura, A. Harada, M. Fukuda, M. Koike, T. Iwatsubo, LRRK2 and its substrate Rab GTPases are sequentially targeted onto stressed lysosomes and maintain their homeostasis. *Proc. Natl. Acad. Sci. U.S.A.* **115**, E9115–E9124 (2018).

22. M. Radulovic, E. M. Wenzel, S. Gilani, L. K. Holland, A. H. Lystad, S. Phuyal, V. M. Olkkonen, A. Brech, M. Jaattela, K. Maeda, C. Raiborg, H. Stenmark, Cholesterol transfer via endoplasmic reticulum contacts mediates lysosome damage repair. *EMBO J.* **41**, e112677 (2022).

23. M. Radulovic, K. O. Schink, E. M. Wenzel, V. Nahse, A. Bongiovanni, F. Lafont, H. Stenmark, ESCRT-mediated lysosome repair precedes lysophagy and promotes cell survival. *EMBO J.* **37**, e99753 (2018).

24. N. Fehrenbacher, L. Bastholm, T. Kirkegaard-Sorensen, B. Rafn, T. Bottzauw, C. Nielsen, E. Weber, S. Shirasawa, T. Kallunki, M. Jaattela, Sensitization to the lysosomal cell death pathway by oncogene-induced down-regulation of lysosome-associated membrane proteins 1 and 2. *Cancer Res.* **68**, 6623–6633 (2008).

25. L. Groth-Pedersen, M. Jaattela, Combating apoptosis and multidrug resistant cancers by targeting lysosomes. *Cancer Lett.* **332**, 265–274 (2013).

26. D. Freeman, R. Cedillos, S. Choyke, Z. Lukic, K. McGuire, S. Marvin, A. M. Burrage, S. Sudholt, A. Rana, C. O'Connor, C. M. Wiethoff, E. M. Campbell, Alpha-synuclein induces lysosomal rupture and cathepsin dependent reactive oxygen species following endocytosis. *PLOS ONE* **8**, e62143 (2013).

27. M. Vila, J. Bove, B. Dehay, N. Rodriguez-Muela, P. Boya, Lysosomal membrane permeabilization in Parkinson disease. *Autophagy* **7**, 98–100 (2011).

28. M. Abu-Remaileh, G. A. Wyant, C. Kim, N. N. Laqtom, M. Abbasi, S. H. Chan, E. Freinkman, D. M. Sabatini, Lysosomal metabolomics reveals V-ATPase- and mTOR-dependent regulation of amino acid efflux from lysosomes. *Science* **358**, 807–813 (2017).

29. J. Behnke, E. L. Eskelinen, P. Saftig, B. Schroder, Two dileucine motifs mediate late endosomal/lysosomal targeting of transmembrane protein 192 (TMEM192) and a C-terminal cysteine residue is responsible for disulfide bond formation in TMEM192 homodimers. *Biochem. J.* **434**, 219–231 (2011).

30. H. Gotzke, M. Kilisch, M. Martinez-Carranza, S. Sograte-Idrissi, A. Rajavel, T. Schlichthaerle, N. Engels, R. Jungmann, P. Stenmark, F. Opazo, S. Frey, The ALFA-tag is a highly versatile tool for nanobody-based bioscience applications. *Nat. Commun.* **10**, 4403 (2019).

31. N. Demaurex, S. Grinstein, "Chapter 16–Measurements of Endosomal pH in live cells by dual-excitation fluorescence imaging", in *Cell Biology: A Laboratory Handbook*, J. E. Celis, Ed. (Academic Press, ed. 3, 2006), pp. 163–169.

32. M. Stagi, Z. A. Klein, T. J. Gould, J. Bewersdorf, S. M. Strittmatter, Lysosome size, motility and stress response regulated by fronto-temporal dementia modifier TMEM106B. *Mol. Cell. Neurosci.* **61**, 226–240 (2014).

33. A. G. Cashikar, P. I. Hanson, A cell-based assay for CD63-containing extracellular vesicles. *PLOS ONE* **14**, e0220007 (2019).

34. C. Stark, B. J. Breitkreutz, A. Chatr-Aryamontri, L. Boucher, R. Oughtred, M. S. Livstone, J. Nixon, K. Van Auken, X. Wang, X. Shi, T. Reguly, J. M. Rust, A. Winter, K. Dolinski, M. Tyers, The BioGRID interaction database: 2011 Update. *Nucleic Acids Res.* **39**, D698–D704 (2011).

35. A. H. Brand, N. Perrimon, Targeted gene expression as a means of altering cell fates and generating dominant phenotypes. *Development* **118**, 401–415 (1993).

36. F. Riedel, A. K. Gillingham, C. Rosa-Ferreira, A. Galindo, S. Munro, An antibody toolkit for the study of membrane traffic in *Drosophila* melanogaster. *Biol. Open* **5**, 987–992 (2016).

37. T. Haller, P. Dietl, P. Deetjen, H. Volkl, The lysosomal compartment as intracellular calcium store in MDCK cells: A possible involvement in $InsP_3$-mediated Ca^{2+} release. *Cell Calcium* **19**, 157–165 (1996).

38. S. H. Barondes, V. Castronovo, D. N. Cooper, R. D. Cummings, K. Drickamer, T. Feizi, M. A. Gitt, J. Hirabayashi, C. Hughes, K.-i. Kasai, H. Leffler, F.-T. Liu, R. Lotan, A. M. Mercurio, M. Monsigny, S. Pillai, F. Poirer, A. Raz, P. W. J. Rigby, J. M. Rini, J. L. Wang, Galectins:A family of animal β-galactoside-binding lectins. *Cell* **76**, 597–598 (1994).

39. G. R. Hammond, M. P. Machner, T. Balla, A novel probe for phosphatidylinositol 4-phosphate reveals multiple pools beyond the Golgi. *J. Cell. Biol.* **205**, 113–126 (2014).

40. J. Zhang, W. Zeng, Y. Han, W. R. Lee, J. Liou, Y. Jiang, Lysosomal LAMP proteins regulate lysosomal pH by direct inhibition of the TMEM175 channel. *Mol. Cell.* **83**, 2524–2539.e7 (2023).

41. J. Li, S. R. Pfeffer, Lysosomal membrane glycoproteins bind cholesterol and contribute to lysosomal cholesterol export. *eLife* **5**, e21635 (2016).

42. H. Appelqvist, L. Sandin, K. Bjornstrom, P. Saftig, B. Garner, K. Ollinger, K. Kagedal, Sensitivity to lysosome-dependent cell death is directly regulated by lysosomal cholesterol content. *PLOS ONE* **7**, e50262 (2012).

43. H. Appelqvist, C. Nilsson, B. Garner, A. J. Brown, K. Kagedal, K. Ollinger, Attenuation of the lysosomal death pathway by lysosomal cholesterol accumulation. *Am. J. Pathol.* **178**, 629–639 (2011).

44. O. B. Davis, H. R. Shin, C. Y. Lim, E. Y. Wu, M. Kukurugya, C. F. Maher, R. M. Perera, M. P. Ordonez, R. Zoncu, NPC1-mTORC1 signaling couples cholesterol sensing to organelle homeostasis and is a targetable pathway in niemann-pick type C. *Dev. Cell.* **56**, 260–276.e7 (2021).

45. P. Gahlot, B. Kravic, G. Rota, J. van den Boom, S. Levantovsky, N. Schulze, E. Maspero, S. Polo, C. Behrends, H. Meyer, Lysosomal damage sensing and lysophagy initiation by SPG20-ITCH. *Mol. Cell.* **84**, 1556–1569.e10 (2024).

46. T. Shima, M. Ogura, R. Matsuda, S. Nakamura, N. Jin, T. Yoshimori, A. Kuma, The TMEM192-mKeima probe specifically assays lysophagy and reveals its initial steps. *J. Cell. Biol.* **222**, e202204048 (2023).

47. S. Aits, M. Jaattela, J. Nylandsted, Methods for the quantification of lysosomal membrane permeabilization: A hallmark of lysosomal cell death. *Methods Cell. Biol.* **126**, 261–285 (2015).

48. U. Repnik, M. Borg Distefano, M. T. Speth, M. Y. W. Ng, C. Progida, B. Hoflack, J. Gruenberg, G. Griffiths, L-leucyl-L-leucine methyl ester does not release cysteine cathepsins to the cytosol but inactivates them in transiently permeabilized lysosomes. *J. Cell Sci.* **130**, 3124–3140 (2017).

49. I. Eriksson, P. Waster, K. Ollinger, Restoration of lysosomal function after damage is accompanied by recycling of lysosomal membrane proteins. *Cell Death Dis.* **11**, 370 (2020).

50. I. Maejima, A. Takahashi, H. Omori, T. Kimura, Y. Takabatake, T. Saitoh, A. Yamamoto, M. Hamasaki, T. Noda, Y. Isaka, T. Yoshimori, Autophagy sequesters damaged lysosomes to control lysosomal biogenesis and kidney injury. *EMBO J.* **32**, 2336–2347 (2013).

51. Y. Zou, M. Stagi, X. Wang, K. Yigitkanli, C. S. Siegel, F. Nakatsu, W. B. Cafferty, S. M. Strittmatter, Gene-silencing screen for mammalian axon regeneration identifies Inpp5f (Sac2) as an endogenous suppressor of repair after spinal cord injury. *J. Neurosci.* **35**, 10429–10439 (2015).

52. J. Schindelin, I. Arganda-Carreras, E. Frise, V. Kaynig, M. Longair, T. Pietzsch, S. Preibisch, C. Rueden, S. Saalfeld, B. Schmid, J. Y. Tinevez, D. J. White, V. Hartenstein, K. Eliceiri, P. Tomancak, A. Cardona, Fiji: An open-source platform for biological-image analysis. *Nat. Methods* **9**, 676–682 (2012).

53. R. Schiemann, A. Buhr, E. Cordes, S. Walter, J. J. Heinisch, P. Ferrero, H. Milting, A. Paululat, H. Meyer, Neprilysins regulate muscle contraction and heart function via cleavage of SERCA-inhibitory micropeptides. *Nat. Commun.* **13**, 4420 (2022).

54. T. Inoue, W. D. Heo, J. S. Grimley, T. J. Wandless, T. Meyer, An inducible translocation strategy to rapidly activate and inhibit small GTPase signaling pathways. *Nat. Methods* **2**, 415–418 (2005); https://doi.org/10.1038/nmeth763.

55. D. M. Shcherbakova, M. Baloban, A. V. Emelyanov, M. Brenowitz, P. Guo, V. V. Verkhusha, Bright monomeric near-infrared fluorescent proteins as tags and biosensors for multiscale imaging. *Nat. Commun.* **7**, 12405, (2016); https://doi.org/10.1038/ncomms12405.
